# From recalcitrance to precision: a robust regeneration, transformation and targeted gene editing framework in *Cajanus cajan*

**DOI:** 10.3389/fgeed.2026.1815812

**Published:** 2026-06-09

**Authors:** Rachana Verma, Jyotsna Bharti, Arulprakash Thangaraj, Sonia Khan Sony, Isha Gupta, Puja Chakraborty, Rashmi Kaul, Bhupendra Rawat, R. Shubhra Maithreyi, Jyoti Priya Samantaray, Sugyan Preet, Kunal Tanwar, Deepak Bhardwaj, Tanushri Kaul

**Affiliations:** 1 Nutritional Improvement of Crops Group, International Centre for Genetic Engineering and Biotechnology (ICGEB), New Delhi, India; 2 Department of Botany, University of Delhi, Delhi, India

**Keywords:** *CRISPR/Cas9*, efficient regeneration, genome editing, phytoene desaturase, transformation

## Abstract

Pigeonpea (*Cajanus cajan* (L.) Millsp.; 2n = 2× = 22) is a drought-tolerant perennial grain legume commonly cultivated in India’s rain-fed and dry land zones, and it is a remarkable natural source of minerals and protein worldwide. Despite decades of research, the constraints associated with tissue culture continue to hinder the genetic improvement of the crop, including the explant’s inability to produce embryogenic calli, direct shoot formation, limited regeneration potential, and a lack of an effective transformation system. Moreover, traditional or molecular breeding approaches for crop improvement is time, resource, and labour-intensive. CRISPR/*Cas9*-mediated approach for targeted trait improvement has emerged as a robust technology for introducing desired genetic modifications in several crop plants. We sought to report an improved protocol for calli production, *in vitro* regeneration, and a *CRISPR-*mediated genome editing of the phytoene desaturase *(PDS)* gene via a biolistic-mediated transformation system in pigeonpea. The indigenously developed construct *(CcPDS_NICTK-2_pCRISPR-Cas9)* harboring pigeonpea codon-optimized *Cas9* and target-specific *sgRNA* was used for transformation in pigeonpea explants (embryonic axis and cotyledonary nodes). The addition of tailored growth regulators and silver nitrate to shoot-induction media boosted plant regeneration to about 86% (±0.04) and transformation efficiency to 46% (±0.04). Sequencing analysis revealed the incurred mutations in the native *CcPDS* gene, with an editing efficiency of approximately 10%. Moreover, this optimized approach can be utilized in the future to generate marker-free genome-edited plants, addressing biosafety concerns and facilitating the acceptance and commercialization of genetically improved crops.

## Graphical Abstract

**Figure:**
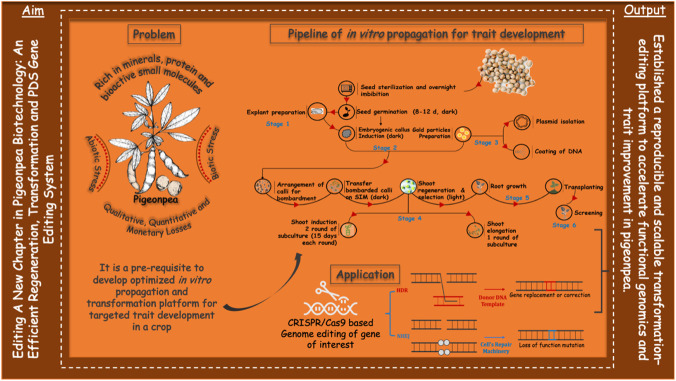

## Highlights

The robustness of this method was significantly attributed to some critical factors:

- Achieved a significant increase in regeneration efficiency through silver nitrate–assisted cytokinin-mediated shoot induction.
- Developed a robust post-bombardment recovery and selection pipeline enabling high-frequency regeneration of edited shoots.
- Targeted gene knockout in pigeonpea using an optimized biolistic-mediated genome editing system.
- Successfully generated PDS-edited pigeonpea events with stable genomic integration of *CRISPR/Cas9* and clearly validated mutation profiles.
- Established a reproducible and scalable transformation–editing platform to accelerate functional genomics and trait improvement in pigeonpea.

## 1 Introduction

Pulses constitute a subgroup of legumes and form an integral part of the human diet and feed for the livestock, majorly because they are an inexpensive source of protein, and an alternative source of carbohydrates, minerals, and vitamins, quintessential for a balanced vegetarian diet (Ferreira et al., 2021; Torheim and Fadnes, 2024). Interestingly, pluses are produced in nearly fifty countries belonging to Asia, Eastern and Southern Africa, Latin America, and some Caribbean countries for umpteen applications (Krishna et al., 2010). As per the report of Food and Agriculture Organisation (FAO Statistical Database, 2019 accessed on 16 January 2024), pigeonpea (*Cajanus cajan* (L.) Millsp.) has retained the position of sixth-best crucial multipurpose consumable grain legume with an area under cultivation up to 5.6 million hectares (Mha), worldwide contribution to global annual production of approximately 4.6 million tons with average productivity 788 kg ha^-1^. Besides, its significance as an enriching source of the dietary elements (Yuniastuti et al., 2021), it is also a remarkable manure and fodder crop. Although there has been an 53.49% increase in the land under cultivation since 1963 (Varshney et al., 2010; FAO Statistical Database, 2022 accessed on 18 January 2024). But due to the lack of knowledge related to technological advancements, insufficient cultivars and poor crop husbandry, pigeonpea production has been consistently decreasing in India. It is also considerably susceptible to several biotic and abiotic constraints including stem canker, *Phytophthora blight, Alternaria blight* (Balai et al., 2023; Singh et al., 2024) significant insect pest *Helicoverpa armigera* (Sharma et al., 2009), *Fusarium wilt*, sterility mosaic (Volp, 2024). Furthermore, the growth and development of this plant are constrained by biotic factors like weed infestation and abiotic stressors such as drought, which ultimately impede its productivity (Upadhyaya et al., 2013). Previous attempts to develop genotypes tolerant to biotic and abiotic factors through conventional breeding methods were unsuccessful due to the limited genetic diversity and incompatibility issues with wild species (Chanda Venkata et al., 2019; Yohane et al., 2022).

The establishment of productive tissue culture and reliable transformation protocols plays a major role in the advent of genetic improvement of the crop plants (Paes de Melo et al., 2020; Lu and Tian, 2022; Guan et al., 2023) and many times recalcitrant plant system too (Galán-Ávila et al., 2020), including pigeonpea. The earlier published reports have established or exhibited the regeneration of shoots from non-meristematic as well as differentiated tissues like a leaf (Singh et al., 2002; Dayal et al., 2003; Asande et al., 2016) and various seedling explants such as hypocotyls, cotyledons (Geetha et al., 1998), cotyledonary nodes (Singh et al., 2003; Jasani et al., 2016), epicotyls (Geetha et al., 1998; Thangella and Fakrudin, 2017) and embryonal axes (Franklin et al., 2000) in pigeonpea. Correspondingly, whole plant regeneration has been shown from different tissue sources of pigeonpea, including the embryonic axis, cotyledonary node, and scutellum (Raut et al., 2015; Karmakar et al., 2019; Kashyap et al., 2020). In Ganguly et al. (2018) showed transformation frequency of 72% in pigeonpea via agrobacterium mediated transformation system using kanamycin as a selection marker for the screening of T_1_ generation. A recent development in genetic transformation via a transgenic approach has facilitated the incorporation of various foreign genes that conferred biotic stress-resistance and nutritional enhancement (Ramu et al., 2012; Ghosh et al., 2017). The most rapidly growing technology is a bacterial monomeric DNA endonuclease called *CRISPR/Cas9* (Clustered Regularly Interspaced Short Palindromic Repeats-associated protein 9), which can be directed to a particular genomic region using a simple 20-base pair (bp) constructed RNA guide sequence, that binds to its target by complementary base pairing (Jinek et al., 2014; Chen et al., 2024). Unlike traditional transgenic methods, which can result in unpredictable insertions and variable phenotypes, *CRISPR/Cas9-*based genome editing offers a precise and targeted approach to crop improvement (Tuncel et al., 2023). This innovative technology preserves the natural genomic architecture, making it a biosafe and reliable tool for introducing desirable traits while minimizing unintended consequences (Chen et al., 2019). CRISPR involves two main components, i.e., a *Cas9* endonuclease and a guide RNA. The *Cas9* is directed to a target site by a site-specific single guide RNA (*sgRNA*), to induce site-specific double-strand breaks (DSBs) in the DNA. Subsequent repair by non-homologous end joining (NHEJ) or homology-directed repair (HDR) introduces targeted modifications, enabling functional gene studies and crop improvement (Belhaj et al., 2015). Phytoene desaturase (*PDS*) is a key enzyme in the carotenoid biosynthesis pathway and is essential for the production of carotenoid pigments required for chloroplast development, photoprotection, and maintenance of photosynthetic activity (Qin et al., 2007). Disruption of *PDS* impairs carotenoid accumulation, resulting in photooxidative damage, chlorophyll loss, and a characteristic albino or photobleached phenotype. Owing to this clear and easily scorable visible phenotype, *PDS* has been widely used as a model target/reporter gene in plant genome-editing studies for rapid validation of CRISPR/Cas9-mediated mutagenesis efficiency (Hooghvorst et al., 2019; Guan et al., 2023; Prasad et al., 2024). Although *CRISPR/Cas9*-mediated genome editing has been reported in major crops such as rice, wheat, maize, sorghum, and tomato, there has been no prior evidence of successful genome editing in pigeonpea through biolistic-mediated transformation (Zhong et al., 2019). In *Cajanus cajan*, Agrobacterium-mediated delivery is feasible, but DNA transfer is often inconsistent and strongly influenced by genotype, tissue response, and regeneration capacity, resulting in variable outcomes. In contrast, biolistic transformation directly delivers *CRISPR/Cas9* constructs or ribonucleoproteins (RNPs) into plant tissues, thereby bypassing host–bacteria interactions and offering several important advantages, including applicability to recalcitrant genotypes, the possibility of DNA-free editing, and transient delivery in embryos or regenerable explants (Hamada et al., 2018). A robust biolistic editing system would therefore be highly valuable not only for routine gene knockout studies but also for future HDR-based applications involving co-delivery of editing vectors and donor templates, as well as RNP-mediated DNA-free genome editing. In addition, from a biosafety and regulatory perspective, the removal of selectable marker genes is desirable because marker-free systems can reduce barriers to commercialization.

In the present study, we established a *CRISPR/Cas9*-based editing workflow in pigeonpea by targeting the *CcPDS* gene using a marker-free, indigenously designed vector delivered through biolistic transformation. Our primary objective was to develop an efficient *in vitro* shoot regeneration and transformation protocol suitable for genome editing in pigeonpea. The construct, *CcPDS_NICTK-2_pCRISPR-Cas9*, carried a plant codon-optimized *Cas9* gene together with *CcPDS*-specific sgRNAs and was introduced into pigeonpea explants by particle bombardment. Successful integration and expression of the editing cassette were validated through molecular analyses, including Sanger sequencing, Southern blotting, and relative gene-expression analysis. The resulting *Ccpds* mutant plants were further characterized through molecular, morphological, biochemical, and confocal microscopic analyses. Collectively, this study demonstrates the feasibility of marker-free biolistic-mediated *CRISPR/Cas9* editing in pigeonpea and expands the available genome-editing toolkit for this crop, with potential utility for future improvement of agronomic performance, stress tolerance, and nutritional quality.

## 2 Materials and methods

### 2.1 Plant materials and explants preparation

Seeds of *Cajanus cajan*, genotype PUSA-992 were procured from the division of genetics, IARI New Delhi. Healthy seeds were sterilized by gentle agitation in 70% ethanol for 3 min, followed by immersion in (0.1% (w/v)) aqueous sodium hypo chloride; NaOCl containing two drops of Tween-20 solution) for 10 min. Finally, these seeds were thoroughly washed several times with double distilled water to ensure the complete removal of sterilizing agents and were then soaked in sterile water for 16–18 h. Pre-soaked seeds, after removal of seed coats, were germinated on half-strength Murashige and Skoog (MS) basal medium (Murashige and Skoog, 1962) with BAP (1 mg/L) at room temperature (27 °C ± 1 °C) under 16 h photo-period, ensuring optimal light and temperature conditions for plant growth.10-days-old grown seedlings were utilized for explants preparation. Embryonic axis (EA) explants were extracted from the basal swollen region of the seedling whereas, cotyledonary nodal (CN) explants were isolated from the apical part of the seedling by incising both cotyledonary leaves and primary apical leaf from the base.

### 2.2 Callus induction

To obtain callus, 450–520 explants of each type (cotyledonary node and embryonic axis) were kept on CIM (callus induction media) with varying combinations of different cytokinins in dark. The basal media used for callus induction comprised of Murashige and Skoog medium (MS) (sourced from HIMEDIA), supplemented with 3% (w/v) sucrose (Thomas Baker, India). Differential doses of cytokinins, viz. kinetin (KN), and zeatin (ZEA) that were tested have been elucidated in (Table 1; Supplementary Material S1; Supplementary Table S1). The experiment was conducted in triplicates and each petri-plate contained 10–12 explants. Calli initiations were obtained after a 10-d-culture of explants on CIM-containing petri-dishes or jam bottles. These were sub-cultured on fresh CIM-containing petri-dishes to obtain an appropriately 1 mm–2 mm sized calli that were ready for transfer to shoot induction media for shoot regeneration.

**TABLE 1:** Effect of various concentrations and combinations of plant growth regulators (PGRs) on embryogenic callus induction via different explants of pigeonpea (CN and EA).

Medium (MS salts +3% sucrose+ 4 g/L Phytagel)	Plant growth regulators (mg/L)		No of explant cultured		Callus induction frequency (%)		Dci	
	Zeatin (ZEA)	Kinetin (KN)	CN	EA	CN	EA	CN	EA
CIM1	0.5	0.1	450	500	26 ± 0.03^a^	22 ± 0.03^b^	20 ± 0.33^a^	18 ± 0.33^b^
CIM2	0.6	0.2	520	410	28 ± 0.06^a^	36 ± 0.04^b^	18 ± 0.33^a^	16 ± 0.33^b^
CIM3	0.7	0.3	510	460	34 ± 0.03^a^	43 ± 0.11^b^	16 ± 0.58^ab^	14 ± 0.33^ab^
CIM4	0.8	0.4	460	480	56 ± 0.03^a^	66 ± 0.04^b^	13 ± 0.33^ab^	12 ± 0.58^ab^
CIM5	1	0.5	470	470	76 ± 0.04^a^	86 ± 0.04^b^	11 ± 0.33^a^	9 ± 0.33^b^

Three replicates of each combination were laid by inoculating 10-12 explants per Jam bottle. Data represent the mean ± SE of three independent experiments of each media combination for each explant, i.e., CN and EA. Values followed by the different superscript letters are significantly different at *P* = 0.05 according to the ANOVA test for the particular media composition. D _ci_: Days to callus induction.

The symbols “a” and “ab” represent statistical significance groupings obtained from multiple comparison analysis. Mean values followed by the same letter are not significantly different, whereas values followed by different letters indicate significant differences at the chosen probability level (p ≤ 0.05).

### 2.3 Shoot regeneration

Initially, for shoot induction different combinations of various cytokines (KN and ZEA), in varying dosages, were utilised. Different combinations of SIM (shoot induction media) incorporating MS media supplemented with a varying dose of cytokinins (KN and ZEA) and silver nitrate (AgNO_3_) were screened for shoot regeneration. The regeneration frequency was obtained by a total number of shoots regenerated from the number of cultured calli (Table 2; Supplementary Material S2; Supplementary Table S2). To enhance shoot length, the regenerated shoots were cultured on a SEM (shoot elongation medium) that consisted of MS media with various doses of gibberellic acid (Table 3; Supplementary Material S3; Supplementary Table S3).

**TABLE 2:** Effect of various concentrations and combinations of plant growth regulators (PGRs) on shoot regeneration via different explants of pigeonpea.

Medium (MS salts +3% sucrose+ 4 g/L Phytagel)	Plant growth regulators (mg/L)			No of calli cultured		Shoot regeneration frequency (%)		D_si_		Shoot length (cm)	
	Zeatin (ZEA)	Kinetin (KN)	Silver nitrate (AgNO3)	CN	EA	CN	EA	CN	EA	CN	EA
A1	0.1	-	0.1	490	490	60 ± 0.12^a^	64 ± 0.08^b^	21 ± 0.33^ab^	20 ± 0.33^ab^	1.8 ± 0.07^a^	2.2 ± 0.06^b^
A2	0.2	-	0.3	460	460	62 ± 0.24^a^	65 ± 0.12^b^	20 ± 0.33^ab^	19 ± 0.33^ab^	2.0 ± 0.06^a^	2.8 ± 0.06^b^
A3	0.3	-	0.5	500	510	64 ± 0.10^a^	66 ± 0.19^b^	18 ± 0.33^a^	19 ± 0.33^ab^	1.9 ± 0.03^a^	2.5 ± 0.03^b^
A4	0.4	-	0.7	500	500	59 ± 0.11^a^	60 ± 0.11^b^	18 ± 0.33^ab^	18 ± 0.33^ab^	1.5 ± 0.09^a^	2.0 ± 0.09^b^
A5	0.5	-	0.9	490	490	57 ± 0.10^a^	61 ± 0.13^b^	18 ± 0.33^ab^	17 ± 0.33^ab^	1.4 ± 0.07^a^	1.9 ± 0.06^b^
B1	0.1	0.1	0.1	470	470	69 ± 0.04^a^	79 ± 0.01^b^	17 ± 0.33^ab^	16 ± 0.33^ab^	3.5 ± 0.07^a^	4.0 ± 0.12^b^
B2	0.2	0.2	0.3	480	480	71 ± 0.11^a^	78 ± 0.08^b^	16 ± 0.33^ab^	15 ± 0.33^ab^	3.6 ± 0.03^a^	4.2 ± 0.12^b^
B3	0.3	0.3	0.5	500	500	76 ± 0.19^a^	89 ± 0.05^b^	16 ± 0.33^a^	15 ± 0.33^b^	3.8 ± 0.06^a^	4.6 ± 0.15^b^
B4	0.4	0.4	0.7	470	470	68 ± 0.10^a^	76 ± 0.01^b^	17 ± 0.33^ab^	16 ± 0.33^ab^	3.4 ± 0.03^a^	4.1 ± 0.15^b^
B5	0.5	0.5	0.9	480	480	65 ± 0.01^a^	74 ± 0.05^b^	16 ± 0.33^ab^	16 ± 0.33^ab^	3.0 ± 0.12^a^	3.9 ± 0.06^b^
C1	0.1	0.1	-	470	450	56 ± 0.11^a^	66 ± 0.04^b^	17 ± 0.33^ab^	16 ± 0.33^ab^	2.7 ± 0.12^ab^	3.1 ± 0.12^ab^
C2	0.2	0.2	-	480	480	54 ± 0.18^a^	68 ± 0.06^b^	17 ± 0.33^ab^	17 ± 0.33^ab^	2.8 ± 0.07^a^	3.2 ± 0.12^b^
C3	0.3	0.3	-	500	500	59 ± 0.26^a^	70 ± 0.17^b^	17 ± 0.33^ab^	17 ± 0.33^ab^	3.0 ± 0.06^a^	3.6 ± 0.09^b^
C4	0.4	0.4	-	440	440	58 ± 0.05^a^	65 ± 0.13^b^	18 ± 0.58^ab^	18 ± 0.33^ab^	2.9 ± 0.12^ab^	3.2 ± 0.12^ab^
C5	0.5	0.5	-	470	490	56 ± 0.16^a^	64 ± 0.17^b^	19 ± 0.33^ab^	18 ± 0.33^ab^	2.8 ± 0.03^a^	3.1 ± 0.09^b^

Three replicates of each combination were laid by inoculating six embryogenic calli (CN and EA) per petri-plate. Data represent the mean ± SE of three independent experiments of each media combination for each explant, i.e., CN and EA. Values followed by the same superscript letters do not show significant difference at *P* = 0.05 according to the ANOVA test for the particular media composition. D _si_: Days to shoot bud initiation.

The symbols “a” and “ab” represent statistical significance groupings obtained from multiple comparison analysis. Mean values followed by the same letter are not significantly different, whereas values followed by different letters indicate significant differences at the chosen probability level (p ≤ 0.05).

**TABLE 3:** The effect of plant growth regulators, GA_3_ (Gibberellic acid) on shoot elongation was investigated.

Medium (MS salts +3% sucrose+ 4 g/L Phytagel)	Conc. Of GA3(mg/L)	Frequency of shoot elongation (%)		Average shoot length (cm)	
		CN	EA	CN	EA
SEM1	0.1	59 ± 0.05^a^	62 ± 0.02 ^b^	4.4 ± 0.06^a^	4.8 ± 0.09^b^
SEM2	0.2	74 ± 0.03^a^	78 ± 0.06 ^b^	5.1 ± 0.09 ^ab^	5.2 ± 0.12 ^ab^
SEM3	0.3	40 ± 0.05^a^	45 ± 0.05 ^b^	4.9 ± 0.09 ^ab^	5.0 ± 0.07 ^ab^
SEM4	0.4	38 ± 0.04^a^	40 ± 0.05 ^b^	4.8 ± 0.07 ^ab^	4.9 ± 0.06 ^ab^
SEM5	0.5	35 ± 0.03^a^	37 ± 0.03 ^b^	4.5 ± 0.06 ^ab^	4.6 ± 0.09 ^ab^

Three replicates of each combination were laid by inoculating five regenerated shoots per jam bottle. Data represent the mean ± SE of three independent experiments of each media combination for each explant, i.e., CN and EA. Values followed by the different superscript letters shows significant difference at *P* = 0.05 according to the ANOVA test for the particular media composition.

The symbols “a” and “ab” represent statistical significance groupings obtained from multiple comparison analysis. Mean values followed by the same letter are not significantly different, whereas values followed by different letters indicate significant differences at the chosen probability level (p ≤ 0.05).

### 2.4 Rooting and transplantation of *in-vitro* regenerated plants

For proper and efficient root initiations, the regenerated shoots were transferred to root regeneration media (RRM) comprised of ½ strength MS media supplemented with differential concentrations of indole-3 acetic acid (Table 4; Supplementary Material S4; Supplementary Table S4). Following the vigorous rooting the plants were transferred to the cocopeat: soil: vermiculite (2:1:2) pots to get acclimatized in the greenhouse conditions that is, a temperature of 27 °C ± 1 °C, and the plants were exposed to natural light supplemented with artificial lighting to maintain a 16-h photoperiod. While to retain humidity the pots were covered with transparent polythene bags for an initial period of 7 days. Following 7 days of hardening transparent covers were removed, and the plants were transferred to the soil pots or greenhouse field.

**TABLE 4:** Effect of plant growth regulator.

Medium (MS salts +3% sucrose+ 4 g/L Phytagel)	Conc. Of IAA (mg/L)	Frequency of root formation (%)	
		CN	EA
RRM1	0.08	58 ± 0.06^a^	60 ± 0.05^b^
RRM2	0.09	70 ± 0.11^a^	72 ± 0.03 ^b^
RRM3	0.1	63 ± 0.3 ^ab^	65 ± 0.03 ^ab^
RRM4	0.2	60 ± 0.05^a^	63 ± 0.03 ^b^
RRM5	0.3	59 ± 0.04^a^	61 ± 0.04 ^b^

IAA: Indole-3-acetic acid on root induction (rhizogenesis) from proliferated shoot. Three replicates of each combination were laid by inoculating five elongated shoots per jam bottle. Data represent the mean ± SE of three independent experiments of each media combination for each explant, i.e., CN and EA. Values followed by the different superscript letters shows significant difference at *P* = 0.05 according to the ANOVA test for the particular media composition.

The symbols “a” and “ab” represent statistical significance groupings obtained from multiple comparison analysis. Mean values followed by the same letter are not significantly different, whereas values followed by different letters indicate significant differences at the chosen probability level (p ≤ 0.05).

### 2.5 CRISPR/Cas9 vector construction and efficient transformation via the biolistic method

A robust marker-free pCAMBIA1300–based binary vector, i.e., *NICTK2-pCRISPR/Cas9* (14.5 kb) was designed indigenously and synthesized (GeneArt: ThermoScientifc, USA). The binary vector harbors a 5.0 kb T-DNA which consists of a 4.4 kb pigeonpea codon-optimized *Cas9 (SpCas9)* gene with two nuclear localization sequences (NLSs) at the proximate ends (Ma et al., 2015). The *NICTK-2_pCRISPR-Cas9* plant transformation vector, which is 14.5 kb in size, includes a 345 bp CaMV 35S promoter and a 175 bp *CaMV* Poly(A) signal terminator. Furthermore, this vector included a multiple cloning site (MCS) with four restriction sites i.e., *BsaI-BsaI, SwaI-SbfI, SwaI-AsiSI, and SbfI-AsiSI* which can be used to integrate single or multiple guide RNAs (*sgRNAs*) for the simultaneous introduction of one or more agriculturally important traits into plants (Supplementary Material S5A; Supplementary Figure S1). To enable *CRISPR/Cas9*-mediated genome editing in pigeonpea, a plant transformation vector was constructed. The backbone of the construct, *NICTK2-pCRISPR/Cas9* (empty vector) (Supplementary Material S5B; Supplementary Figure S1), contained the *Cas9* coding sequence driven by the *CaMV* 35S promoter and terminated by the *CaMV* Poly(A) signal. The vector was propagated in *E. coli* Top10 competent cells and isolated followed by screening with P35S and Cas9 primers (Supplementary Material S6A; Supplementary Table S5). This vector was used to establish stable integration and expression of *Cas9* in transformed pigeonpea plants. Furthermore, to establish effective gene editing system we have chosen the Phytoene desaturase (*PDS*) gene as a proof-of-concept. We targeted the *PDS* gene, a well-established marker for *CRISPR/Cas9* editing, which is involved in chlorophyll biosynthesis. The *Cajanus cajan PDS* gene (reference Sequence no: XM_020357799.2; LOC id 109797685 accessed on 4 February 2024) having exon count 13 were retrieved from NCBI database (Supplementary Material S6B). To ensure efficient editing of the *CcPDS* gene, *sgRNA* target sequences 20 bp long and situated just upstream of a 5′-NGG-3′ PAM site (5′-GAT​CAT​ATT​CAG​TCC​TTG​GG-3’) were picked employing CRISPR RGENE software (www.rgenome.net, accessed on 11 March 2024). The selected sgRNA had a GC content of 50%. Potential off-target sites were initially assessed using CRISPR-P v2.0 and further predicted using Cas-OFFinder implemented through CRISPR RGEN Tools (www.rgenome.net, accessed 11 on March 2024), based on sequence similarity against the *Cajanus* reference genome (Supplementary Material S7; Supplementary Figure S4). We cloned the sgRNA expression cassette in *Bsa1-Bsa1* restriction sites harbouring *AtU3b* promoter (Accession No. X52629) and terminators, respectively and was subsequently cloned in the intermediary pMA-RQ entry vector. Finally, the developed *sgRNAs* expression cassette was digested from the pMA-RQ entry vector and eventually cloned into *NICTK-2_pCRISPR-Cas9* for developing final plant transformation *CcPDS_NICTK-2_pCRISPR-Cas9* vector (Supplementary Material S5C). Successful cloning and orientation of the *sgRNA* cassette were confirmed prior to plant transformation by utilizing *PDS1* Cons primers and PUbi primers (Supplementary Material S6A; Supplementary Table S5). The designer construct was propagated in the *E. coli* and harnessed via the method described by Sambrook (Sambrook et al., 1989) for biolistic transformation. Successively, for nucleic acid delivery with a PDS-1000 He (Bio-Rad, Hercules, Calif.) DNA coated gold particles were prepared by enumerating 50 μl CaCl_2_ (1M), 20 μl spermidine (0.1 M) and 5 µg plasmid DNA in the 50 µl aliquot of gold particles (1 µm diameter, 30 mg/ml) which was mixed via vortexing at 4 °C. To recollect the DNA coated gold particles the mixture was centrifuged and the pellets of DNA coated gold particles were washed with absolute alcohol, followed by re-suspension in 30 μl of absolute ethanol. Approximately 20 to 25-days-old actively growing embryogenic calli (CN and EA) were subjected to transformation and about 50–60 embryogenic calli (CN and EA) were clustered in the centre of the CIM5 containing petri-dishes. A vacuum of 22 inches of Hg (74.5 kPa), rupture disk rated for a pressure of 1,100 psi (7.58 MPa), and a 6 cm distance of the plant tissue from the stopping plate was maintained in each experiment. After bombardment, the calli were maintained in the existing petri-dish for 3 days in the dark, followed by 15 days in light to accomplish proper multiplication and growth. The regenerated shoots were transferred to elongation media, followed by 2-3 subcultures. Later, the regenerated shoots were transferred to RRM and profusely rooting plants were hardened and post acclimatization shifted to soil pots or fields in the greenhouse. This visual representation illustrates the key stages of *in-vitro* shoot regeneration and biolistic-mediated transformation in pigeonpea, providing a comprehensive understanding of the protocol.

### 2.6 PCR-based confirmation of transgenes

For the analysis of gene integration, a polymerase chain reaction was carried out on total genomic DNA (gDNA) extracted from young green leaves of 30-day-old putative transformants (T_0_) and wild type (WT) by the modified cetyl trimethyl ammonium bromide (CTAB) method (Sambrook et al., 1989). Wild-type control plants used for phenotypic and molecular comparison were regenerated through the same tissue culture protocol as the transformed plants, but without CRISPR/Cas9 construct-mediated editing. The genomic DNA (gDNA) of putatively transformed T_0_ plants were analyzed employing Cas9 specific primer, forward primer (5’-TTC​GAC​CAG​TCC​AAG​AAC​GG-3’), and the reverse primer (5’ CTT​GAC​CTT​GGT​GAG​CTC​GT-3´), where gDNA of WT plants and plasmid *Cc_PDS NICTK2-pCRISPR/Cas9* was used as a negative and positive control, respectively. The reaction was carried out on a thermal cycler with optimized cycling condition at 94 °C for 4 min followed by 30 cycles at 94 °C for 55 s, 58 °C for 45 s, 72 °C for 1 min with a final extension at 72 °C for 8 min. Furthermore, out of these *Cas9* positive transgenic events, were taken for further PCR analysis employing additional *CcPDS* gene specific primers as mentioned in the (Supplementary Material S6A; Supplementary Table S5). The amplified fragments were excised and purified using the QIAquick Gel Extraction Kit (Qiagen, USA), and subsequently sent for sequencing. To trace the pattern of inheritance of the exogenous DNA in the T_1_ generation, every twenty T_1_ seeds were germinated from individual T_0_ (*CcpdsP88, CcpdsS11, and CcpdsS51*) plants, and PCR analysis was carried out as mentioned above. Mutation analysis was initially performed in 30-day-old T0 plants and was further validated in the T1 generation. Twenty seeds from each individual T_0_ plant (CcpdsP88, CcpdsS11, and CcpdsS51) were germinated, and PCR analysis was performed as described above. The purified products were further assessed via a *T7EI* assay, adhering to the manufacturer’s protocol (New England Biolabs, USA) with the designated primer pair as mentioned in (Supplementary Material S6A; Supplementary Table S5).

$$\text{Transformation Efficiency}=\frac{\text{Total}\;\text{no}.\;\text{of}\;\;\text{plants}\;\;\text{showing}\;\;\text{integration}\;\;\text{of}\;\;\text{Cas}9\;\;\text{gene}}{\text{Total}\;\;\text{no}\;.\;\text{of}\;\;\text{calli}\;\;\text{used}\;\;\text{for}\;\;\text{transformation}}\;x\;100$$

### 2.7 Quantitative real-time PCR analysis of *Cas9* and *sgRNA* expression

Total RNA was extracted from fully expanded leaves of transgenic (T_0_ and T_1,_) and wild-type pigeonpea plants (30-days-old) using TRIzol reagent (Invitrogen, USA) following the manufacturer’s protocol. RNA concentration and purity were assessed spectrophotometrically (A260/A280 ratio), and RNA integrity was verified by agarose gel electrophoresis. First-strand cDNA synthesis was performed using the SuperScript™ III Reverse Transcriptase kit (Agilent Technologies, USA) with 1 µg of total RNA in a 20 µL reaction volume. The *Cajanus glyceraldehyde-3-phosphate dehydrogenase* (*GAPDH*, reference Sequence no: XM_020355692.2; LOC109796067 accessed on 29th March 2026) were used as an endogenous positive control in all real-time PCR assays (Supplementary Material S6D). *GAPDH* was selected based on previous validation of housekeeping genes in pigeonpea, where it was identified as one of the most stable reference genes for gene-expression studies (Sinha et al., 2015). Gene-specific and *Cas9, sgRNA* and *GAPDH* primers were designed using Primer3 (NCBI) and are listed in Supplementary Material S6A (Supplementary Table S5). The primers designed for the *Cas9* gene generated a 124 bp amplicon, whereas the *GAPDH* reference gene primers produced a 95 bp amplicon. For *sgRNA* expression analysis, a forward primer was designed from the target-specific guide sequence and a reverse primer was designed from the conserved gRNA scaffold region generating a 92 bp amplicon. Quantitative real-time PCR (qRT-PCR) was carried out using Brilliant II SYBR® Green QPCR Master Mix (Agilent Technologies, USA) in an Agilent Real-Time PCR Detection System (Agilent Technologies, USA). The thermal cycling conditions were as follows: initial denaturation at 95 °C for 5 min, followed by 40 cycles of 95 °C for 10 s and 60 °C for 30 s in a final reaction volume of 25 µL. Melt-curve analysis was performed at the end of amplification to confirm primer specificity and the absence of non-specific products. *Cas9* transcript levels and *sgRNA* were calculated using the 2^−^ΔCt method with *GAPDH* as the internal reference. Each experiment included three biological replicates and three technical replicates per sample. Data are presented as mean ± SD, and statistical significance was determined using Student’s t-test at p ≤ 0.05.

### 2.8 Southern blot analysis

To perform southern hybridization 20 µg of genomic DNA from both WT and randomly selected T_1_ plants was digested with *EcoRV* restriction enzyme and electrophoresed in 0.8% agarose gel which was utilized to transfer the resolved DNA to a nylon membrane (Hybond-N+, Amersham, UK) by stacking it overnight. The PCR amplified *Cas9* gene which was labelled with dig provided in DIG High Prime DNA Labelling and Detection Starter kit was used as a probe. Pre-hybridization, blocking, and detection of blots were performed following the manufacturer’s instructions (Roche PCR DIG Probe synthesis and DIG Luminescent Detection Kit: Sigma-Aldrich, Switzerland). In the present analysis, DNA from WT pigeonpea plant was used as a negative control.

### 2.9 Quantification of pigments concentration

To assess the efficiency of *CRISPR/Cas9*-mediated targeted mutation and its impact on chlorophyll and carotenoid production in *CcPDS-*edited pigeonpea lines, pigment quantification was performed. Fresh leaf tissue from the transgenic lines and wild type plants was harvested, followed by grounded in the liquid nitrogen and promptly immerse the ground samples in 10 mL of DMSO. Extraction of chlorophyll were conducted at 60 °C in complete darkness for 4–5 h to prevent degradation. Subsequently samples were allowed to reach room temperature followed by centrifugation at 10000 rpm for 10 min. Carefully supernatant were taken and the absorbance of extracted chlorophyll was determined with a spectrophotometer (PerkinElmer) at 663 and 645 nm, and the total chlorophyll (a+b) concentration in the extract was calculated according to the equation: concentration of chlorophyll (mgL^−1^) = 20.2 × absorbance at 645 nm + 8.02 × absorbance at 663 nm. The absorbance of carotenoids was determined at 480 nm and concentration was calculated according to equation= (A480 nm + (0.114 x A663) - (0.638x A645) x V/1,000 x W. Tissue chlorophyll and carotenoids content was expressed as milligrams per gram dry weight.

### 2.10 Chlorophyll autofluorescence

Small sections of 3 to 4-week-old leaf tissue from wild-type (WT) plants (∼8–10 mm^2^) and from the *PDS* mutant plants (pale green/partial albino and albino) were mounted on glass slides using adhesive bandages, with the basal side facing upward. A ×40 water immersion objective lens was used to observe the autofluorescence of chlorophyll. The samples were excited by 1% laser power at 488 nm, and chlorophyll autofluorescence was detected in the 680–750 nm range. Images were captured using the Olumpus Fluoview FV3000 confocal system and FV315-SW software (https://www.olympus-lifescience.com/). The captured images (z-stacks) were postprocessed using Cellsens software (https://www.olympus-lifescience.com/en/software/cellsens/).

### 2.11 Statistical analysis

In this study, each experiment was replicated thrice to collect the data. The data generated was statistically analysed using the MSTAT-C program. Statistical significance was determined at 5% and 1% probability levels. In addition, means were compared by the critical difference (CD at P = 0.05, 0.01) for each dataset.

## 3 Results

This manuscript presents a reliable and optimized protocol for *in vitro* shoot regeneration, biolistic-mediated transformation, and establishment of a genome-editing platform in pigeonpea.

### 3.1 Optimizing callus induction in *Cajanus cajan* explants using different cytokinin combinations

**FIGURE 1:**
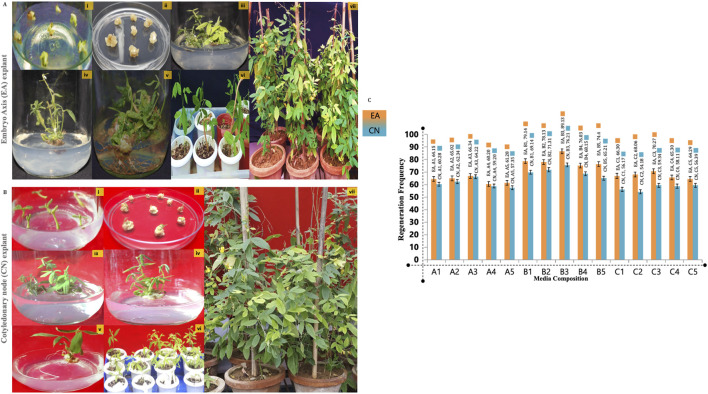
**(A i–vii).** Regeneration of multiple shoots from embryonic axis explants derived from in vitro-germinated seedlings of pigeonpea. **(i)** Embryonic axis explants at day 0 cultured on MS medium supplemented with 0.5 mg/L kinetin and 1 mg/L zeatin for callus induction (CIM 5). **(ii)** Induction of callus on CIM after 8 days. **(iii)** Initiation of shoot regeneration on 0.3 mg/L ZEA+ 0.3 mg/L kN + 0.5 mg/L AgNO_3_ (SIM). **(iv)** Shoot elongation on MS medium supplemented with 0.2 mg/L GA_3._ (SEM). **(v)** Response of regenerated shoot for root induction on ½ MS containing 0.09 mg/L IAA (RIM). **(vi)** Acclimatization of plantlets in plastic pot containing mixture of cocopeat:soil: vermiculite. **(vii)** *In vitro* produced plants in the glass house showing normal growth, flower production, and pods that contain viable seeds. **(B i–vii)** Regeneration of multiple shoots from cotyledonary node explants derived from in vitro-germinated seedlings of Pigeonpea. **(i)** Cotyledonary node explants at day 0 cultured on MS medium supplemented with 0.5 mg/L kinetin and 1 mg/L zeatin for callus induction (CIM). **(ii)** Induction of callus on CIM after 12 days. **(iii)** Initiation of shoot regeneration on 0.3 mg/L ZEA +0.3 mg/L kN + 0.5 mg/L AgNO_3_ (SIM). **(iv)** Shoot elongation on MS medium supplemented with 0.2 mg/L GA_3._ (SEM). **(v)** Response of regenerated shoot for root induction on ½ MS containing 0.09 mg/L IAA (RIM). **(vi)** Acclimatization of plantlets in plastic pot containing mixture of cocopeat:soil: vermiculite. **(vii)** *In vitro* produced plants in the glass house showing normal growth, flower production, and pods that contain viable seeds. **(C)** Schematic representation of shoot regeneration frequency in pigeonpea via embryonic axis (EA) and cotyledonary node (CN) explants. For efficient shoot regeneration in the two chosen explants, calli were kept under various concentrations and combinations of ZEA and KN plant growth hormones, whereas, AgNO3 (0.1–0.9 mg/L) was incorporated in the SIM to test the outcome on shoot regeneration frequency. MS basal medium augmented with KN 0.3 mg/L, ZEA 0.3 mg/L along with AgNO_3_ 0.5 mg/L was seen to be the best for the proliferation of multiple shoots. Effective shoot regeneration frequency from embryonic axis explants (86%), and by cotyledonary node explants (76%). Data represent the mean ± SE of three independent replicated of each media combination for each explant.

Surface-sterilized seeds cultured on half-strength Murashige and Skoog (MS) basal medium supplemented with 1.0 mg/L 6-benzylaminopurine (BAP) exhibited a germination efficiency of approximately 90%–95%. In contrast, seeds cultured on full-strength MS medium showed lower germination rates, and the resulting seedlings were weak and less suitable for subsequent regeneration. This indicates that ½ strength MS medium offers a more favourable physiological condition for pigeonpea seed germination. Supplementation with BAP (1.0 mg/L) further enhanced seedling vigor, producing robust explants with characteristic swelling at the embryonic axis and hypocotyl base. Ten-day-old seedlings were selected to prepare embryonic axis (EA) and cotyledonary node (CN) explants (Figure 1Ai,Bi).

Unlike previous studies that commonly utilized combinations of auxins and cytokinins, this study focused on optimizing cytokinin-only treatments to enhance callus induction potential. Various hormonal combinations of kinetin (KN) and zeatin (ZEA) were tested with MS basal medium. Among these, medium CIM5 containing 0.5 mg/L kN and 1.0 mg/L ZEA produced the highest callus induction frequency callus initiation occurred within 8 days for EA explants and 12 days for CN explants (Figures 1Aii,Bii). The frequency of callus formation was highest i.e., 86% in embryonic axis explants compared to 76% in cotyledonary node explants (Table 1). These results highlight that the type and concentration of plant growth regulators are critical for maximizing *in vitro* response and callus production efficiency.

### 3.2 *In vitro* regeneration system for pigeonpea

To optimize shoot regeneration, various combinations of cytokinins (ZEA and KN) were evaluated, along with different concentrations of AgNO_3_ (0.1–0.9 mg/L) as a supplement in the shoot induction medium (SIM) (Table 2). The best response was observed using MS medium supplemented with 0.3 mg/L KN, 0.3 mg/L ZEA, and 0.5 mg/L AgNO_3_, resulting in prolific multiple shoot formation. The embryonic axis explants showed a regeneration frequency of 89%, while cotyledonary node explants achieved 76% (Supplementary Material S6; Supplementary Table S2). Shoot bud initiation occurred within 15–20 days of transferring embryogenic calli onto SIM (Figure 1Aiii,Biii). Initial shoots often exhibited yellowing and stunted elongation, prompting the optimization of gibberellic acid (GA_3_) levels for proper elongation (Table 3). The addition of GA_3_ at 0.2 mg/L significantly enhanced shoot elongation, suggesting that excess cytokinins can inhibit this process (Figure 1Aiv,Biv). Elongated shoots (up to 3.0 cm) were subcultured onto fresh medium to minimize phenolic buildup. For root induction, elongated shoots were transferred to root induction medium (RIM) containing 0.09 mg/L indole-3-acetic acid (IAA), resulting in a 72% rooting efficiency (Table 4; Figure 1Av,Bv). Acclimatized plantlets, transferred to vermiculite pots and later to soil, developed into phenotypically normal plants with healthy leaves (Figure 1Avi,vii,Bvi,vii). The regenerated plants were morphologically identical to seed-derived progeny, producing normal flowers and fertile pods with viable seeds (Supplementary Material S57; Supplementary Figure S2).

### 3.3 Genome editing vector development and identification of the target *PDS* gene sequence

**FIGURE 2:**
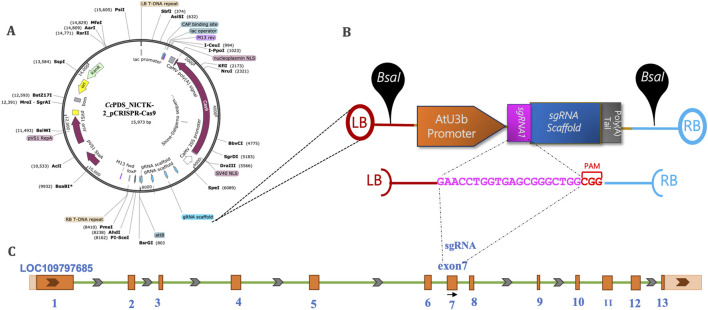
Designing and development of the *CRISPR/Cas9*-based plant transformation vector for the incorporation of the desired mutations. **(A)** Represents the *CcPDS_NICTK2_pCRISPR-Cas9* plant transformation. **(B)** sgRNA cassette with target sequence and PAM highlighted in red. **(C)** Graphical representation of the *PDS* genes and the exonic region. The number of exons has been described below each box representing an exon. The single sgRNAs were designed from the exon7. The 5’ and 3’ UTR region of *PDS* gene is highlighted in beige color, exons in orange and green lines represent the introns.

The target region for *CcPDS* gene was identified by examining the reference genome of *Cajanus cajan* using the *Arabidopsis thaliana* phytoene desaturase (*AtPDS*) gene sequence as a query. Thirteen *CcPDS* exons were retrieved from the NCBI database, revealing a single-copy gene located on chromosome 1, with a coding sequence of 1,743 bp encoding a 570-amino acid protein. The genomic region (7,498 nucleotides) comprises 13 exons and 12 introns. Sequence alignment and phylogenetic analysis confirmed conserved regions suitable for *sgRNA* design (Supplementary Material S7; Supplementary Figures S3, S5). The *CcPDS_NICTK-2_pCRISPR-Cas9* plant transformation vector (Figure 2A) harboured *Cas9* protein under *CaM35S* promoter for efficient translation *in vivo*. Along with *Cas9* it harbours the *sgRNA* cassette (Figure 2B) targeting the exon seven region as the target sequence (Figure 2C).

### 3.4 Embryogenic axis explant is more amenable for biolistic mediated transformation

The regenerated plants from embryogenic calli derived from embryonic axis (EA) and cotyledonary node (CN) explants, transformed with *NICTK-2_pCRISPR-Cas9* (empty vector; Supplementary Material S5A; Supplementary Figure S1) were screened for *Cas9 i*ntegration. PCR amplification of the expected 535 bp *Cas9* fragment was detected in 243 of 260 regenerated EA plants and 215 of 225 regenerated CN plants, whereas no amplification was observed in WT controls, confirming successful transgene integration (Supplementary Material S7; Supplementary Figure S6). Although CN explants exhibited a higher regeneration frequency (89%) compared with EA explants, transformation efficiency was greater in EA-derived explants (up to 46%) than in CN explants (39%). Collectively, these findings establish EA explants as the more efficient system for genetic transformation in pigeonpea (Supplementary Material S7; Supplementary Figure S7). To establish CRISPR/Cas9-mediated genome editing in pigeonpea, the endogenous *PDS* (*phytoene desaturase*) gene was targeted. Based on their superior regeneration and transformation competence, a total of 658 embryogenic calli derived from EA explants were subjected to biolistic transformation using the *CcPDS_NICTK2_pCRISPR-Cas9* vector (Figure 2). Following bombardment, calli were incubated in the dark for 20 days to facilitate proliferation and enhance cellular plasticity prior to regeneration.

### 3.5 Molecular characterization and expression analysis of *Cas9* transgenic pigeonpea

**FIGURE 3:**
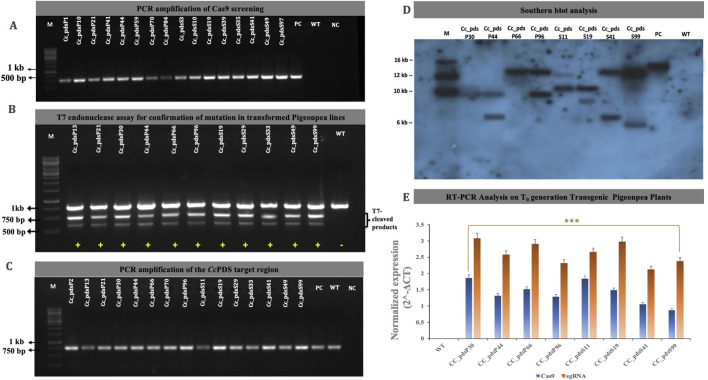
Molecular characterization of putative positive transformed T_0_ plants **(A)** Primary validation is confirmation of *Cas9* gene via PCR amplification. **(B)** Confirmation by T7 endonuclease in *Cas9* positive lines. PCR amplified target from control (WT) and transformed lines (1-11) were subjected to T7E1 assay. The edited lines showed the target fragmented into two bands, which were not present in the WT plants. Genomic DNA of pigeonpea leaves derived from screened *Cas9* positive lines, lanes 1–16 represent positive *Cas9* gene pigeonpea transformed lines. **(C)** Amplification of 750 bp of the *CcPDS* target region. **(D)** Representative Southern blot analysis of established T_0_ transformants showing the single copy number of *Cas9* gene in screened transgenic plants. These observations were obtained by the digestion of genomic DNA from eight transgenic plants using restriction enzyme *EcoRV* and Cutsmart two buffer obtained from NEB. Lane M 100 ng DNA molecular weight marker III, Digoxigenin labelled, and lane PC five ug positive control plasmid DNA. **(E)** The normalized/relative expression of the *Cas9* gene and sgRNA cassette was analyzed in T_0_ plants in comparison with wild-type plants, the Relative expression values are presented as −log10(2^−^ΔCt), where higher values correspond to lower transcript abundance. Total RNA was extracted from one-month-old plants grown under standard conditions, followed by cDNA synthesis and quantitative expression analysis. The experiment was conducted in triplicate, and the data are presented as mean ± standard deviation (SD) (n = 3). M 1 kb DNA ladder (Thermofisher), WT genomic DNA of untransformed pigeonpea plant, PC positive control (plasmid DNA template), NC negative control. + represent edited lines; - non-edited (wild type).

**FIGURE 4:**
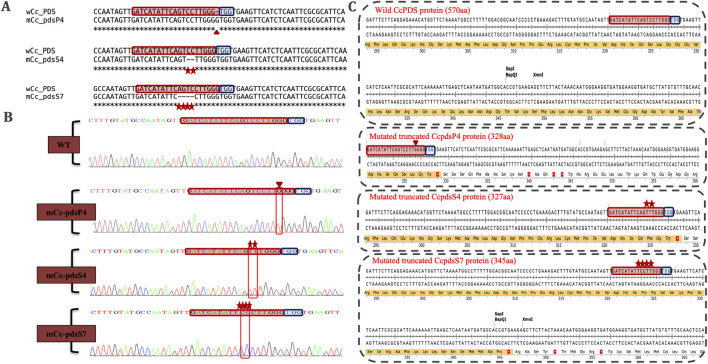
Sequence based identification of mutation generated by *CcPDS_NICTK2-pCRISPR/Cas9* construct. **(A)** Represents the sequence alignment between wild PDS gene sequence and edited plants mCcpdsP4, mCcpdsS4 and mCcpdsS7. **(B)** Chromatograms of the edited plants in comparison with the wild *PDS* gene sequence. **(C)** The insertion of a single G in mCcpdsP4 sequence resulted in generation of pre-mature stop codon translating into truncated protein of 328 amino acids (aa), deletion of CC from mCcpdsS4 sequence resulted in generation of pre-mature stop codon translating into truncated protein of 327aa and deletion of AGTC from mCcpdsS7 sequence resulted in generation of pre-mature stop codon translating into truncated protein of 345aa. sgRNA sequence has been highlighted with Red box, PAM sequence has been highlighted with blue box, insertion mutation has been indicated with red triangle and red star represents the deletion mutation.

To confirm the presence of the *Cas9* transgene in pigeonpea transformants, genomic DNA was isolated from putative T_0_ transgenic plants, wild-type (WT) plants used as a negative control, and the plasmid vector used as a positive control. PCR analysis was performed on 566 transformed plants, of which 303 regenerated plants exhibited the expected 535 bp amplicon corresponding to the *Cas9* gene (Figure 3A; Supplementary Material S7; Supplementary Table S7). No amplification was detected in WT samples, confirming the specificity of the assay and successful gene integration (Figure 3B; Supplementary Material S6A; Supplementary Table S5). A second round of screening was conducted using T7 endonuclease, which revealed the presence of additional bands in 67 *Cas9-*positive transformed plants compared to the wild-type plants (Figure 3C). However, the remaining edited lines were not digested by *T7E1*. Furthermore, Southern blot analysis was conducted to assess transgene integration copy number. Genomic DNA digested with *EcoRV* (single site within construct) revealed distinct hybridization patterns indicating both single- and double-copy insertions, with fragment sizes ranging from approximately 6–16 kb. 67 lines showed hybridization signals for the *Cas9* gene when probed with a DIG-labelled *Cas9* probe, whereas no hybridization signal was detected in WT samples (Figure 3D). These results revealed successful establishment of *Cas9* gene into the genome of pigeonpea. These putative positive plants were analyzed via sanger sequencing to validate these results (Figures 4A,B).

### 3.6 PDS gene mutation generates pre-mature stop codon in pigeonpea

**FIGURE 5:**
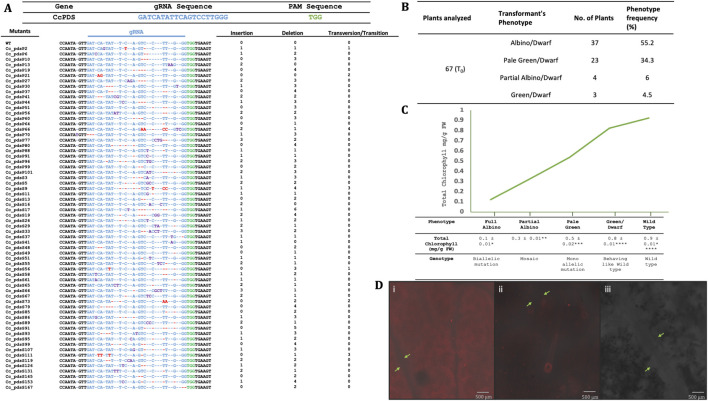
**(A**) The table represent mutation data of the edited plants along with the details for type of mutations observed. Where blue-colored nucleotides represent the sgRNA sequence, while green nucleotides represent the PAM sequence, dark blue nucleotides represent the insertion mutations, red dashed represents deletion mutations and red nucleotides represents the substitution mutations. **(B)** The sixty-seven edited plants exhibited four distinct phenotypes; the data represents the phenotype frequency. **(C)** Pigment quantification analysis for four mutant phenotype and wild type Pigeonpea plants, Data represent the mean ± SE of independent experiments of chlorophyll quantification. Values followed by the asterisk shows significant difference at P = 0.05 according to the ANOVA test. **(D)** Autofluorescence of chlorophyll in pigeonpea leaves. **(i)** WT leaf of pigeonpea showing autofluorescence of chlorophyll (red) (excitation at 488 and 561 nm, emission at 500–569, 570–700 nm). **(ii)** The weak fluorescence in PDS, CcpdsS7 mutant showing pale green phenotype **(iii)** No fluorescence in PDS, CcpdsP4 and CcpdsS4 showing albino phenotype. The white arrows shows the veins of the leaf. Scale bar = 500 µm.

**FIGURE 6:**
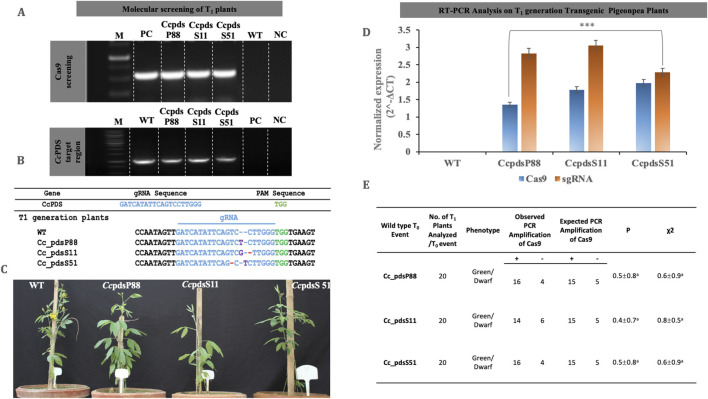
Molecular characterization of putative positive transformed T_1_ plants **(A)** PCR amplification of 535 bp and 750 bp of *Cas9* gene and *CcPDS* target region from T_1_ positive plants, respectively. **(B)** Target region sequence of the CcPDS gene from the edited pigeonpea plants of T_1_ generation. **(C)** WT and edited T1 plants showing phenotypic differences. **(D)** The Normalized/relative expression of the *Cas9* gene and sgRNA cassettte was analyzed in T_1_ plants in comparison with wild-type plants. The experiment was conducted in triplicate, and the data are presented as mean ± standard deviation (SD) (n = 3), the relative expression values are presented as −log10(2^−^ΔCt), where higher values correspond to lower transcript abundance. **(E)** Inheritance of Cas9 gene in T_1_ plants of three mutation events that showed green phenotype df = 1; P = 0.05; χ2 = 3.841. Data represent the mean ± SE of independent experiments of segregation test. M 1 kb DNA ladder (Thermofisher), WT genomic DNA of untransformed pigeonpea plant, PC positive control (plasmid DNA template), NC negative control.

*CRISPR/Cas9*-mediated editing of the *PDS* gene resulted in a mutation efficiency of 10.01%, calculated as the number of edited plants with confirmed mutations in the target region relative to the total number of bombarded calli (Supplementary Material S9; Supplementary Table S7). Mutation analysis was initially performed in T_0_ plants at the (30-day-old) and was further validated in the T_1_ generation. Targeted regions were validated by Sanger sequencing and chromatogram-based sequence analysis. Sequence analysis revealed diverse mutation types, including a single G insertion, a two-nucleotide (CC) deletion, and longer deletions (e.g., AGTC) upstream of the PAM site within the *CcPDS* target region (Figure 4C). These indels generated premature stop codons, leading to truncated proteins, whereas non-edited plants retained the full-length 570–amino acid protein. Additional events exhibited insertions/deletions (indels) and base substitutions (transitions and transversions) within the *CcPDS* locus (Figure 5A). The spectrum of mutations resulted in three distinct phenotypic classes: complete albino, partial albino, and pale-green plants (Figure 5B; Supplementary Material S7; Supplementary Figure S8). Certain events, including *CcpdsP88* and *CcpdsS11*, displayed nucleotide alterations but retained protein structure similar to wild-type with a green phenotype (Figure 6A; Supplementary Material S7; Supplementary Figure S9). Another event, *CcpdsS51*, exhibited nucleotide and predicted protein sequence modifications yet displayed a green dwarf phenotype. Structural prediction using AlphaFold2 indicated no major alteration in overall protein conformation (87% confidence) (Supplementary Material S7; Supplementary Figure S10). The T1 progeny of selected edited events retained the mutations and exhibited the corresponding dwarf green phenotype together with continued Cas9 expression, confirming stable inheritance of the edited alleles.

### 3.7 Effects of *PDS* gene mutation in pigment accumulation

Since *PDS* gene have vital role in pigment synthesis, therefore, we quantified the Chlorophyll a, b, total chlorophyll and carotenoid contents in mutated and wild type plants with regard to the concentration of chlorophyll content through spectrophotometry as well as confocal microscopy (Figures 5C,D). As expected, three distinct mutant plants revealed a remarkable reduction in the chlorophyll a and b and total chlorophyll followed by carotenoid contents in contrast to the wild type leaf indicating the disruption of *PDS* gene. Furthermore, the images of autofluorescence for WT clearly displays presence of dense concentration of chlorophyll in WT pigeonpea leaf (excitation at 488 and 561 nm, emission at 500–569, 570–700 nm) (Figure 5Di). Whereas, the weak fluorescence in mutants, i.e., *CcpdsP4* (pale green phenotype) showing partial albino phenotype and in *CcpdsS7* (Chimeric event) (Figure 5Dii). Furthermore, no fluorescence in *CcpdsS4* (No Chlorophyll) showing full albino phenotype suggesting disruption of the *CcPDS* gene which leads to affect the chlorophyll biosynthesis pathway (Figure 5Diii). This indicated that the confocal microscopy results were in concurrence with chlorophyll estimation assay via spectrophotometry.

### 3.8 Expression analysis of *Cas9* and *sgRNA* in transgenic plants of T_0_ and T_1_ generations

The expression of the *Cas9* gene was analysed in eight randomly selected T_0_ transgenic pigeonpea plants along with wild-type plants using quantitative real-time PCR (qRT-PCR) (Figure 3E; Supplementary Material S10; Supplementary Tables S8, S9), This analysis was further extended to the T_1_ generation to assess the stable inheritance and continued expression of the transgene in the subsequent generation (Figure 6D). Transcript abundance was normalized against the endogenous reference gene *GAPDH* to account for variation in cDNA input and amplification efficiency. The analysis revealed clear accumulation of *Cas9* transcripts in the positive transgenic plants, whereas no *Cas9* transcripts were detected in the wild-type plants, as expected. These results confirmed stable expression of the *Cas9* transgene in the transformed pigeonpea plants. Furthermore, *sgRNA* expression was analysed in both T_0_ and T_1_ plants (Figures 3E, 6D; Supplementary Material S10; Supplementary Tables S10, S11). The detection of *sgRNA* transcripts in both generations further confirmed the stable expression of the *CRISPR/Cas9* editing cassette in the transgenic pigeonpea lines. Since Cas9 and *sgRNA* transcripts were not detectable in wild-type plants, expression values were represented as log-transformed 2^−^ΔCt values normalized to *GAPDH*. Relative expression values are presented as −log10(2^−^ΔCt), where higher values correspond to lower transcript abundance.

### 3.9 Inheritance of mutation in next-generation

To assess the inheritance of the edited alleles and transgene in the next-generation, the T_1_ progeny of CcpdsP88, CcpdsS11, and CcpdsS51 were analysed. PCR analysis confirmed the presence of the Cas9 transgene in the T_1_ plants (Figure 6A), while Sanger sequencing showed that these plants harboured the same mutations as those detected in the corresponding T_0_ generation (Figure 6B). Although the T_1_ mutants exhibited a green phenotype, they showed dwarf stature and delayed flowering in comparison with wild-type plants (Figure 6C). Segregation analysis of the Cas9 transgene in the T_1_ progeny of the green mutant lines demonstrated transmission to the next-generation following a Mendelian 3:1 inheritance ratio (Figure 6E). Together, these findings confirm the stable inheritance of the edited alleles and demonstrate the successful establishment of a functional and heritable CRISPR/Cas9 genome-editing platform in pigeonpea.

## 4 Discussion

It can be inferred from the earlier literature that no proper, well-reproducible tissue culture based transformation techniques have been fully established in pigeonpea. Pigeonpea, a vital legume crop, has long faced significant yield and production losses due to abiotic and biotic factors. Despite numerous attempts to enhance its yield and nutritional quality through genetic transformation, previous methods have been hindered by low transformation frequencies. This limitation is primarily attributed to the crop’s recalcitrant nature, scarce regenerable tissues, and poor *in vitro* rooting (Ghosh et al., 2014a; Ghosh et al., 2017). However, the transformation frequencies registered in those methods were not noteworthy because of the recalcitrance displayed by this legume crop (Atif et al., 2013; Ghosh et al., 2014b). The scarce number of regenerable tissues, for agrobacterium-assisted gene transformation and along with that, low regeneration frequency contributes to the failure in continuing selection treatments. Thus, inadequate regeneration in tissue culture-based methods; the frequent collapse of shoots following selection, and the troublesome *in vitro* rooting manifested to be significant obstacles to transgenic development in pigeonpea crop. These problems ingeniously compelled the need for the development of a reliable plant tissue culture-based transformation method in pigeonpea. In the initial phase of our research, we established a highly efficient *in vitro* regeneration system for pigeonpea, a crucial prerequisite for successful genome editing. Across this study, it was found that several parameters, including type and composition of basal medium salt, plant growth regulators as well as their doses used at various stages, are critical factors influencing the regeneration frequency. Ten-day-old germinated seedlings were found suitable for the explant’s preparation. Embryonic axes from 10-day-old seedlings exhibited superior morphogenic competence, consistent with reports highlighting developmental stage as a critical determinant of *in vitro* regeneration (Gahan and George, 2008; Supplementary Material S7; Supplementary Figure S7). Notably, a cytokinin-only regime (kinetin + zeatin) supported high callus induction and shoot organogenesis, eliminating the requirement for exogenous auxin. In the present work, a hormonal mixture of kinetin (0.5 mg/L) with zeatin (1 mg/L) contributed to the highest calli induction efficiency (86%) in Pigeonpea (Table 1). A previous report demonstrated shoot initiation in pigeonpea from explants kept on appropriate doses along with several combinations of plant growth regulators (PGRs) such as MS basal media supplemented with 0.5 mg/l BAP, 0.1 mg/l NAA 0.05 mg/l thidiazuron (TDZ) (Thangella and Fakrudin, 2017), 3.0 mg/l BAP, and 0.5 mg/l NAA (Jasani et al., 2016; Figures 1A,B). Unlike other studies, our approach addresses the common problems of inconsistent callus formation and low regeneration efficiency by utilizing a refined balance of kinetin and zeatin without requiring exogenous auxins. These results corroborated with earlier reports that indicated improved callus formation on a medium engineered with a combination of several cytokinins rather than using auxin-cytokinin (Franklin et al., 2000). During our study, we found MS medium fortified with 0.3 mg/l ZEA and 0.3 mg/l kN led to remarkable shoot regeneration frequency (89%) (Table 2). Our approach employed lower cytokinin levels, ensuring improved shoot development while maintaining high callus induction rates. As pulses contain a sufficient amount of endogenous auxin, callus initiation could be achieved even when auxin is not added in the medium, thus, optimized concentrations were essential for maximizing regeneration efficiency. In addition to zeatin and kinetin, the addition of silver nitrate promotes multiple shoot proliferation, presumably by adsorbing phenolic compounds and suppressing ethylene activity in the culture medium, thereby improving shoot formation (Davoudipahnekolayi et al., 2024). We found consistent result and multiple shoot proliferation by supplementing SIM with 0.5 mg/l AgNO_3_ (Figure 1C; Table 2).

Collectively, these refinements overcame key morphogenic bottlenecks that historically limited pigeonpea transformation. Regenerated pigeonpea shoots showed enhanced elongation on MS medium containing 0.2 mg/L GA3 in our experiments (Table 3). Likewise, other researchers have communicated results wherein, the additions of GA3 alone or in combination with BAP lead to efficient shoot regeneration as well as elongation (Srivastava and Raghav, 2014; Kumar et al., 2016). Successful establishment of the seedlings from the regenerated shoots depends on the development of proper roots. In the present study, we have demonstrated 72% of the regenerated shoots, flourishingly rooted on the RRM (Table 4). Correspondingly, complete plant regeneration has been shown from different tissue sources of pigeonpea, including the embryonic axis, cotyledonary node, and scutellum (Raut et al., 2015).

In the second phase of this study, we successfully established a *CRISPR/Cas9*-mediated genome editing protocol in pigeonpea using biolistic-mediated transformation. CRISPR/Cas9 has transformed the field of genome editing, offering an effective solution for trait improvement in various organisms and crops (Ferreira and Reis, 2023; Zhu et al., 2020). Gene editing protocols are well-established in several legume species for instances in *Medicago* (Wolabu et al., 2020), Pea (Guan et al., 2023), lotus (Wang et al., 2019), cowpea (Che et al., 2021), pigeonpea (Prasad et al., 2024; Senthil et al., 2025) and soybean (Cai et al., 2020). Most of these crops showed gene editing which relies on agrobacterium-mediated transformation while biolistic delivery facilitates the introduction of diverse molecular cargo, including plasmid DNA, single-stranded DNA (ssDNA), RNA, and ribonucleoproteins (RNPs) assembled from *in vitro* transcribed (IVT) components and recombinant proteins (Hamada et al., 2018; Yuan et al., 2024). Earlier reports in pigeonpea via agrobacterium-mediated transformation have used different tissue sources like shoot apices and cotyledonary nodes for direct regeneration as well as those that show indirect regeneration from callus derived from embryonic axes has been elucidated (Geetha et al., 1999). Karmakar et al. reported the use of a non-tissue culture-based method to obtain the transformation efficiency of 83% from embryonic axis-attached cotyledons (Karmakar et al., 2019). Low transformation frequencies from 13.7% to 32% have been shown in T_1_ generation using embryogenic calli obtained from different explants via agrobacterium-mediated transformation (Babaoglu et al., 2000; Ramu et al., 2012). It can be inferred from the earlier literature that no proper well-reproducible tissue culture-based transformation techniques have been fully established in pigeonpea. Based on previous reports embryonic axis, followed by a cotyledonary node has been shown as the most reproducible and rapid tissue for transferring advantageous traits in legumes (Babaoglu et al., 2000; Swathi Anuradha et al., 2008). The current protocol develops up to high regeneration frequency in case of the untransformed calli (76%), transformed calli generated (49%), and higher transformation efficiency up to 46% using biolistic-mediated transformation (Table 5). The transformation efficiency achieved in our study (∼46% using biolistic-mediated CRISPR/Cas9 transformation) is substantially higher than previously reported efficiency of 15.2% by Prasad et al. (2024) and 9.16% by Senthil et al. (2025) in pigeonpea. Our study made use of the biolistic-mediated delivery system, which makes it easier to directly introduce CRISPR/Cas9 components into tissues (Figure 2). Southern blot analysis confirmed stable, predominantly low-copy *Cas9* insertions, while transcriptional profiling demonstrated consistent expression in T_0_ lines (Figure 3). Low-copy integration is often associated with stable expression and reduced silencing risk. The transformation efficiency achieved in our study (∼46% using biolistic-mediated CRISPR/Cas9 transformation) is substantially higher than previously reported efficiency of 15.2% by Prasad et al. (2024) and 9.16% by Senthil et al. (2025) in pigeonpea. Our study made use of the biolistic-mediated delivery system, which makes it easier to directly introduce *CRISPR/Cas9* components into tissues. In genome editing of *Cajanus cajan*, biolistic transformation offers practical advantages over Agrobacterium-based systems, particularly given the crop’s well-known recalcitrance. While Agrobacterium-mediated aforementioned protocols have been reported, they often remain highly genotype- and tissue-dependent, with variable DNA transfer efficiency and frequent issues such as explant browning or poor regeneration. In contrast, the biolistic approach circumvents host–bacteria compatibility constraints and eliminates reliance on vir gene–mediated T-DNA delivery. When combined with highly regenerable explants such as embryonic axes and an optimized regeneration regime, carefully balanced cytokinins along with additives like silver nitrate to improve morphogenic response, particle bombardment has demonstrated improved transformation and editing efficiency. Moreover, the capacity to directly deliver *CRISPR/Cas9* constructs or RNP complexes provides added flexibility, including the possibility of generating transgene-free edits. Collectively, these features make biolistic-mediated transformation a robust and adaptable option for achieving reliable genome editing even for homology donor repair (HDR) mechanism and plant recovery in challenging legume species like pigeonpea (Bharti et al., 2025).

**TABLE 5:** Transformation efficiency of *Cas9* gene in putative transgenic pigeonpea plants.

Medium (MS salts +3% sucrose+ 4 g/L Phytagel)	No of seeds innoculated	Total no. of calli used for transformation	No. of plants regenerated	Plant regeneration frequency (%)	No. of plants expressing Cas9 genes	Transformation efficiency (%)
Embryonic axis	714	527	260	49 ± 0.27^a^	243	46 ± 0.25^a^
Cotyledonary node	710	535	225	42 ± 0.32^b^	215	40 ± 0.06^b^

Data represent the mean ± SE of three replicate for each explant, i.e., CN and EA. Values followed by the different superscript letters shows significant difference at *P* = 0.05 according to the ANOVA test for each explant.

The symbols “a” and “ab” represent statistical significance groupings obtained from multiple comparison analysis. Mean values followed by the same letter are not significantly different, whereas values followed by different letters indicate significant differences at the chosen probability level (p ≤ 0.05).

Further, to establish the *CRISPR/Cas9* system in pigeonpea we have selected the phytoene desaturase (*PDS*) gene, and employed the prior develop regeneration and transformation protocol. The primary aim to choose the *PDS* gene as it plays a key role in carotenoid biosynthesis, with mutations causing photobleaching or albinism that can be visually detected without specialized assays (Komatsu et al., 2020; Siddappa et al., 2023). Consequently, *PDS* serves as a popular visual indicator for evaluating *CRISPR/Cas9* efficiency in various plant species (Bánfalvi et al., 2020; Komatsu et al., 2020). In order to generate efficient mutations, we have designed an optimal single gRNA(sgRNA) based on CRISPR RGENE software from CcPDS gene to target exon seven in Pigeonpea (Figure 2). The seventh exon was chosen for gene editing due to CRISPR-P analysis showing that it provided optimal gRNAs starting with 'G', which are suitable for use with the U3 promoter to achieve successful editing outcomes. Furthermore, a comparison of PDS genes from dicot and monocot plants revealed conserved sequences, which were used to design single guide RNAs (Supplementary Material S7; Supplementary Figure S5). Preliminary screening of transformed plants using gene-specific *Cas9* primers was followed by a second screening via T7 assays, which revealed the presence of an extra band in the 67 edited lines compared to the wild-type plants. Similar results were previously reported in chilli using T7 endonuclease cleavage, which revealed additional DNA fragments in *CaPDS-*edited lines (Figure 3B), indicating successful genome editing (Bulle et al., 2024). The sequence analysis revealed that most mutations have insertions or deletions (indels), deletions of two/more nucleotides from the upstream of the PAM region. Numerous studies have documented the prevalence of small insertions and deletions (indels) resulting from *CRISPR/Cas9*-mediated genome editing in various crop species (Hussain et al., 2018; Mainkar et al., 2023). Edited lines harbouring the *CcPDS* construct, exhibited single nucleotide insertions (1bp G insertion), leading to chlorophyll-deficient pale-green phenotypes, as exemplified by *CcPDS-P4* (Figures 4A,B). Our results are consistent with a previous report demonstrating the efficacy of *CRISPR/Cas9*-mediated mutagenesis in several plant species with a single nucleotide polymorphism (SNP) facilitated efficient genome editing (Gao et al., 2017; Odipio et al., 2017). Sequence targeted random deletions in transgenic pigeonpea lines were confirmed (2bp CC) and (4bp AGTC) deletion, exhibiting partial to full albino phenotypes (Figure 4C).

The molecular expression data further strengthen the interpretation of the editing results. Cas9 transcripts were consistently detected in transformed T_0_ plants and remained detectable in the T_1_ generation, while no amplification was observed in wild-type plants (Figure 3E). Likewise, sgRNA expression was detected in both T_0_ and T_1_ plants, confirming continued presence and transcriptional activity of the editing cassette across generations (Figure 6D). These data, together with stable Cas9 expression in the progeny, indicate that the CRISPR/Cas9 components remained transcriptionally active after transmission to the next-generation. At the same time, the identical target-site mutations recovered in T_1_ plants demonstrate that the edits were heritable and not merely transient somatic events. In addition, we have quantified the Chlorophyll a, b, total chlorophyll and carotenoid contents from the district phenotypes (pale green, partial and full albino) along with wild type plants to determine the effect of *CcPDS* mutations. We found a substantial reduction in partial and fully albino shoots in comparison to pale green and wild type. The reduction in pigment content shows a functional knockout of the *CcPDS* gene resulted in generation of pre-mature stop codon translating into truncated protein (Figure 4C). As the expression of the *PDS* gene is directly linked to chlorophyll production, and considering no additional copy of this gene is present in pigeonpea genome, therefore chlorophyll concentration was used to relate to the mutation pattern of the genome-edited plants. Plants exhibiting a fully albino phenotype for instance *CcpdsS4* showed no traces of chlorophyll, indicating edits in both alleles and thus biallelic mutants, which were further confirmed by sequencing (Figures 4B,C). Similar findings were reported earlier, where a significant reduction of photosynthetic pigments was observed by disruption of onion *AcPDS* gene (Mainkar et al., 2023). As anticipated, the edited plants displayed an albino phenotype due to impaired chlorophyll production, rendering them unviable for seed production and precluding the collection of seeds for subsequent generations. The *PDS* gene knockout via CRISPR reduces gibberellin production, causing stunted growth and a dwarf phenotype by disrupting chlorophyll, carotenoid, and gibberellic acid biosynthesis (Siddappa et al., 2023). Furthermore, chlorophyll concentration analysis of *CcpdsP4* and other mutants displaying a pale-green phenotype suggested a monoallelic mutant genotype, where one allele retained the wild-type sequence and the other carried mutations, as confirmed by sequencing analysis (Figure 4B). In contrast, chlorophyll-deficient partial albino mutants such as *CcpdsS7* exhibited mosaic genotypes (Figure 5A). Although *in vitro* propagation can generate chimeric events due to regeneration from both transformed and untransformed meristematic cells, our platform produced a very low chimeric frequency (6%), highlighting the efficiency of the optimized protocol (Figure 5B). The confocal microscopy results were consistent with the chlorophyll estimation assay further confirming the mutation phenotype correlation (Figure 5D). Two events, *CcpdsP88* and *CcpdsS11*, carried nucleotide modifications that did not translate into protein changes, as shown by *in silico* translation of their mutated sequences. Additionally, one mutant, *CcpdsS51*, displayed modifications at both the nucleotide and protein levels; however, the only amino acid substitution was at position 325, where serine (neutral, polar) was replaced by leucine (neutral, non-polar). Structural prediction with AlphaFold2 (Jumper et al., 2021) indicated that this S325L substitution did not affect the overall protein structure. The predicted model, with ∼87% confidence, showed strong structural similarity between wild-type *CcPDS* and the *CcpdsS51* mutant protein (Supplementary Material S7; Supplementary Figure S10). These mutants exhibited a green phenotype, likely due to the preserved tertiary structure, whereas their dwarf stature may be attributed to altered codon usage bias affecting translation efficiency (Wu et al., 2025). Finally, the successful transmission of edited alleles and the Cas9 transgene in a Mendelian 3:1 segregation pattern in the T_1_ generation confirms that the system developed here is functionally heritable (Figure 6E). Although fully albino mutants were not recoverable for seed production because of their severe physiological impairment, the green dwarf T_1_ progeny derived from selected events retained the edited genotype and showed continued Cas9 expression. Taken together, the off-target analysis, molecular validation, pigment quantification, and inheritance data provide a coherent line of evidence that the phenotypes recovered in this study were caused by specific editing of *CcPDS*. This study demonstrates the effectiveness of *CRISPR-Cas9* in targeting specific plant genes, providing valuable insights for future agricultural and plant biology research. The implication of pigeonpea codon-optimized *Cas9* harboring *CaMV 35S* promoter and terminator, single guide RNA (*sgRNA*) expression displayed enhance editing efficiency along with mutation frequency as demonstrated in various crops such as pea (Guan et al., 2023), soybean, rice (Wang et al., 2016), and *M. truncatula* (Wolabu et al., 2020). The editing efficiency of 10% surpasses (Table 6) the editing efficiency for *PDS* reported by Prasad et al., 2024 which was 4%–6% and the 8.80% editing efficiency documented by Senthil et al., 2025. This highlights the superior performance of our optimized transformation and regeneration protocol in enhancing both gene transfer and editing outcomes. Key methodological variations, including the transformation approach and the optimized regeneration protocol, can be responsible for the observed variation in editing efficiency. In recent years, genome editing smartly and precisely has transformed plant breeding and modifying legumes by the introduction of desirable traits that shows resistance to abiotic and biotic stress breaking the barriers over transgenic or conventional breeding methods (Singh et al., 2024; Sita et al., 2017). Furthermore, the development of *CRISPR–Cas* systems have revolutionized plant genetics and breeding by enabling precise genome editing through technologies like base editing and prime editing, which can introduce single-base changes with high accuracy (Molla et al., 2021). The efficient protocol discussed in the earlier studies opens the door for utilizing other *CRISPR-Cas*-based genome editing tools like Cas12a, base editors, and prime editors, offering powerful options for advancing genome editing-based breeding in pigeonpea. Thereby, in future the present approach could be utilized for generating marker-free genome edited plants that will reduce the concern of biosafety regulations and acceptance of commercialization of crops.

**TABLE 6:** Editing efficiency of *Cas9* gene in transgenic pigeonpea plants.

Medium (MS salts +3% sucrose + 4 g/L Phytagel)	Total no. of calli used for transformation	No. of plants regenerated	Plant regeneration frequency (%)	No. of plants expressing Cas9 genes	Transformation efficiency (%)	No. of plants horbouring mutations	Editing efficiency (%)
Embryonic axis	658	566	86.03 ± 0.35	303	46.07 ± 0.21	67	10 ± 0.02

Data represent the mean ± SE of three replicate for EA explant. Values followed by the different superscript letters shows significant difference at *P* = 0.05 according to the ANOVA test for each explant.

## 5 Conclusion

This study successfully demonstrates the biolistic-mediated *CRISPR/Cas9* system in pigeonpea achieving albino-phenotype mutants. Here, we built up a strong *in-vitro* plant regeneration system using embryonic axis and cotyledonary node as explants with substantial enhanced transformation efficiency. This reproducible, speedy, compatible protocol overcomes significant hurdles associated with the genetic improvement of the crop. Plant codon optimized *Cas9,* paired with *CaMV 35S* regulatory elements and sgRNA, boosts gene editing efficiency and mutation rates. This breakthrough enables gene function exploration and agronomic trait advancement in legume crops, efficient *CRISPR/Cas9* gene editing in these species.

## Data Availability

The original contributions presented in the study are included in the article/Supplementary Material, further inquiries can be directed to the corresponding author.
