# Supplementary material for: From recalcitrance to precision: a robust regeneration, transformation and targeted gene editing framework in Cajanus cajan
Source: Front Genome Ed. 2026 Jun 9;8:1815812. doi: 10.3389/fgeed.2026.1815812 (PMC13287048; doi:10.3389/fgeed.2026.1815812)
Supplement: Supplementary file 2 [file Supplementaryfile6.docx]

**Supplementary file 6 A: Table S5.** List of primers used in this study

| **Primer Name** | **Forward Primer** | **Reverse Primer** |
| --- | --- | --- |
| **Cas 9 primer** | 5’ TTCGACCAGTCCAAGAACGG 3’ | 5’ CTTGACCTTGGTGAGCTCGT 3’ |
| **PUbi primer** | 5´ TCTAACGGACACCAACCAGC3´ | 5´CCCCAAAGAGAAACACTGGC 3´ |
| **P35S primer** | 5´ AGCGTGTCCTCTCCAAATGA 3´ | 5´GATGTGAACATGGTGGAGCA 3´ |
| **Cc_PDS primer** | 5´ ATCAGGACTGAATGCAACCC 3´ | 5´ TCCCTTTCCAGTTGTCAGGT 3´ |
| **PDS1_Cons** | 5’TGCAGCCTGTGATGGATAACT 3’ | 5´ ACTCGGTGCCACTTTTTCAAG 3´ |
| **Cc SgRNA_F:** | **5’GATCATATTCAGTCCTTGG 3’** | **5’ CGACTCGGTGCCACTTTTTC 3’** |
| **Cc_GAPDH ( glyceraldehyde-3-phosphate dehydrogenase)** | **5’ ATGGCATTCCGTGTTCCTAC 3’** | **5’ CCTTCAACTTGCCCTCTGAC 3’** |

**Supplementary file 6 B:** The *Cajanus* PDS (Phytoene desaturase) gene (reference Sequence no: XM_020357799.2; LOC id 109797685) were retrieved from NCBI database.

**LOC109797685 15-cis-phytoene desaturase, chloroplastic/chromoplastic [ *Cajanus cajan* (pigeon pea) ] Exon count 13 (size: 7497 bp)**

ATTTTAAGAATTGTTGTCGTGATCTTCTTCATCATCCACCAGAGAATATTCAAACACACCGCACCAGACA

CCACTCCTCTCCTCTGCTGCTTTCTCCACCAAACTCACTTTCTCTCACTCTCAATCCAATGGCCGCATGT

GGCTGCATATCTGTCTCCAACTTCAATTGCCTCCTTGGCGCCAGAAAACTATCCAAATTCGCTTCTTCAG

ATGCCACACTTTCCCTATCATTCGCTGCGAGCGACTCCATGGGTCTTACTCTGCGACCCACTTCAACTCT

TTCTCCTAAGAGAAACTTTTTCTCTCCCTTGCGCGTCGTTTGCGCCGATTATCCTCGCCCGGAGCTCGAA

AACACCGCCAATTTCATCGAAGCTGCTTACTTATCTTCCACGTTTCGTGCTTCTCCGCGGCCACAGAAAC

CCTTGAAGGTCGTTATTGCCGGTGCAGGTAATTAACGAACTATGTCTGTACTCTATACCCATTTCGTTTT

ATGCTCCAAAATTGTGTCTTTGCTACTAAATTTTGTGTTTTTATTGCTTTAGTCGTTCTGGGTGGTTTCA

GATTAGTACCTTTCATCAACAAGTTGTTGGTCCGACAAGTCCTAACAATAACATGGTTATAAAAAATGAA

TACTTCGTGCTAATAAATTAAAAAAAAAAAATAGGACCCGTTAGGATTTTCTAGTGCATCATCTTTTTAG

AATTTCTTTTCTCCTTTTTCTTCTAGCTTGATGTTTGTTTGAAAGTTGTTGGGCTTTGGAACTTAGAAGC

CTGATTCCTTGGGCTAGGCATTGGGAGTTCCTTTCTTTTCGATGAAATTGAAGTATATACTTTTGATTAC

ATCGTGAGTCTTTGACAGTTTGGTGTCAATAACGATAAAAATAATGGGATTTTACCATCTTCAATTTTTT

AGTGAAATTTCTGATTAAGGTTACCCTGATATCATGTTGGATATTTAAAACAAAAGCGTTTGTGAGCTAA

TCTTTGAATTTTTGATCTGTGTCTCTTGGATTGATGTATGTTTCGAATTATCTCAGGATTGGCTGGTTTA

TCAACTGCAAAGTATTTGGCTGATGCTGGGCATACGCCTATATTGCTGGAAGCAAGAGACGTTCTAGGTG

GAAAGGTTTTCCTGCTAATTTAATCCCATTTGTCAATAAGTTGTCACTTTGCAAATTCTGTTCATCGTTT

TAGCCTGCTGTTTAATTAAATAAGTAGTTTGTTCTCGTGTTGTTATATCATAATCTAATGGATATATTGG

CTTTATATGATCTATAATTTGAATTCTTGTCTTCTGAGTCAATTTTACTCGACTTTGGAATGGTCATTTT

GAAGTTTATCACCAGGCTTGTTGGAGATTACCACAGTACATTCAACTTATTTATTATAATTTATAAATTG

ATTTGAATCCATTGTGTTTTTATGCTATTATTTCATTTTCTAAGGTTGCTGCTTGGAAAGACAAGGATGG

AGACTGGTATGAGACTGGCCTGCACATCTTCTGTAAGTTCATTAAAATCTCAGGCTTAACTTTTTTTATG

TCATACATTTATTGAACCTATTTCCGATAAAGAGGATTTAAAGCTAGATGATCAGGAATCTTCCTAGAGC

TACTAAAGAAACTATTGAGATTTGATCCAGATTTCCATCTCAAATTTAATCCAGATTTCCATATGAGATT

TGTTCCTGATTTCCATTTGATCTCTATCATTATTATTTCATATTTCTGTTTTTATTATTCTTGTTAAGTT

ACCATTTTATTGTATTTAATGTCTGATTGTATAATCCTATAATCAATCATTAAGTCTGATATTCAAGTGA

AACCACTCAGTAAAGGAAGTAATAAACAAAGGTCATAACCATGCTTTGGTCTTCTCCTAATAGCTTAAGC

TATTGATAGAGTTGGTCCTCAACACTTGGTCACAGCCTTTCGCTTCGTGATCAAGTGGTTGGAATTTTAA

TCCTTGTTGCCCCACTCTTTTGATTAACAATTAAATTTCAGCAAACAGGTTTGGGTTAACTTATGCTCTG

TCCAAGCTTCAAGCTCAAATCAATGGGCCTTTGAATGAGCGGGCTTTTTGGCCTCTTAACATTGCTTTTG

GATGAGCTAGCGCTTGGCACACATATTGAACTATTTTGATATATCTTTTTAGTTTTTCTTTCTAACTGAA

TCATTTACTTGCATTAAAATTTCCCCCCTCCCTACAGTTGGGGCTTACCCTAATGTGCAGAACCTATTTG

GTGAACTTGGCATTAATGATCGGTTACAATGGAAGGAGCATTCCATGATTTTTGCTATGCCAAATAAGCC

TGGAGAGTTTAGTCGATTTGATTTTCCTGATGCCTTTCCCGCCCCATTAAATGGTAAGATGCAAGACTTA

ATGTATGTGTGCATATTTAAATACCTACCTGAAGTTTGATGTCTCTGTAAAAAGCACGTTGAATGCCTTC

TGTCTTTAGAAAACCTACAGTTTGGAATTTGCATCTGTGTGCTGCAGAATGTGTAATACTAACTAGCTAT

TCTCCTGCCTGTGTTATGGGTAGTGCCTTGAAGTTGAAAACCAGAATTCTGATTAAAAGGATGTTTAGTT

TCGTTGAAAGTTCAAACGCAATACTCCAGTCAAATTTTGATTTTGAATATATCTTAATGCATTTGATAAT

CGTTGGAACAAGTTATGTTAAATCAGTTCCAGTATAGAACAAGCTAAACACAATATGCCAGTCAAATTTT

GATTTTGATTACATCTTAATGCATTTGATAATCATTGAAACAAGATGTGCTAAATTGGTTCCATTTTATG

TAAGTTGAAAATTAAAAATTGTAAACTCAAAAACAGAACATGCGTTAAATTTGTTCCCTTTAAATTAAAA

AATTCAACATCAAACAGAGAACAAGCATTTGGTGTTTAGCTTGATCTGTACTCTCTTATTTCTATTTCTT

TCTTCACTAATCTGGTTTGCAGGACAGGAACATATTTGTCTCTTTCAAAAAGAACTAACTGATGCTTAAA

TTCTTCATTTTTATTTAATATCCGAGTGATTCCCTTTCTATGATTTTGTTTCATTATGCTTTACCTTCTA

AATGTTAACCAGTATTTACAAGTTGCATGCCATGAATGTGATAACAGGAATATGGGCAATATTGAGGAAC

AATGAGATGCTGACATGGCCAGAGAAAGTCAAATTTGCAATTGGGCTTCTGCCAGCTATGCTTGGTGGAC

AACCATATGTTGAGGCTCAAGATGGTCTTTCTGTTAAGGAATGGATGGCAAGACAGGTATAGAACTTGTC

TTGATTGTGTAACCTTAGCAGTCCATAAATAGATTTAACTAACCTTTGCTACACCCATATTTTTCTTCTG

TCATCCCTTCAAAGCCCAACGTTCACTACCATTGAGTATAGTCAGACACATCTCAGTAAAATAAAATTTC

CATTTGAGCCTGATAGGTACTTTGTGATCACAAATAACCCATGCTCCATAGTCGGTTTTAAAGGACTCCA

ACTCTATCATAGACAATGTGGTGGAATTTTTGTTTTGATTCTTTCTGATATTCTCATTGTAAAATGAGTC

AATTGTAGAAAATTTCTCTGTTTATTTTTCCTGTTTTCTCTGCTAAAATTTCTTCTTTTATTTTTGACCT

AAAAAAATCTTTATAGAAAAAAAATGGAAGTACATTACTAAAACAGGTATTGCGTTACTATTTATAAAAT

TAAACTCCCCTTCTAGACTTGACTTTATACGTACTGATGCTTATTGTCTTCCTGGTTACTCAGGTATTTA

ATTTTTTTAGCACAATAAGAAAAAGAAATGAATAAGGAAAATTGGTTTTAAAAAAGGGAAGATAAAGAAG

AAATAATTTCTTTCTCTTCCCCCATTCATATAAGGAGGGTTGTCTATGTCTGCATGCCTTTATTAGCATA

GTTGATTCATTTGCTATTATTGAAAGAAAAATCAAACATGGGAGACAAACTGGGATATAGTTGAAGTTGT

TTGTTTAGGTTTGGCTTGTACCATTATTGAAATACTAGTCATTGTGATCTGGATTGATGTGAAGAAGGGG

CTTCTGTGCTCTAAGAATGGGAGAATAAATTAATTTTGTATATTAGATTTTGCTTTGTCTCTTAATTTAA

TGATGACAGTTGAGTATTTTACTTTACTGGAAAATATAGAAAGGATATCATAGTTGTAGTTGTTAGATTG

TATTACTTGATATTTACGTAATGCTTTAAAATATATTTCATCAGGACTGAATGCAACCCTCTCATGGTCT

GTATTGCAAATAATTTGAGAATAACAAAATGTTTTCTCCTTATTGTAGGGAGTACCTGAACGAGTGACTG

AAGAGGTGTTCATAGCAATGTCAAAGGCACTAAACTTTATCAATCCTGATGAACTTTCAATGCAATGTAT

ATTAATTGCTTTAAACCGATTTCTTCAGGTGTGATCATTTCCTTTCATTAAACACTATTCCAATGGCATT

ATTGTGTGATTTTTTAGGTCATTTCAATCTCATTATAACAGTTGAGTGGAATCAGATTAGTAAGCTCTAA

GATGGCCTTTTATTTCACAAACATGTTTTGTGGAACTTGGGATCAATTTTTTCCTTCAACTAATTGTGAT

ATTGTTAAATTCTTGAATAGGAGAAACATGGTTCTAAAATGGCCTTTTTGGACGGCAATCCCCCTGAAAG

ACTTTGTATGCCAATAGTTGATCATATTCAGTCCTTGGGTGGTGAAGTTCATCTCAATTCGCGCATTCAA

AAAATTGAGCTCAATAATGATGGCACCGTGAAGAGCTTCTTACTAAACAATGGGAAGGTGATGGAAGGTG

ATGCTTATGTGTTTGCAACTCCAGGTATTTTTTTTTCTTATTTTCCCACTGTGTTAATTAATTGTTTTCT

TGGCATCTTAATCTCCATAACATTTTTGAGGAATTTCCACCCTTGAAAACAGTGGATATTCTGAAGCTTC

TTCTACCTGACAACTGGAAAGGGATTCCATATTTCCAGAGATTGGATAAATTAGTTGGAGTCCCAGTCAT

TAATGTTCACATATGGTAAGTAACTGCTTTTGATGTTGGAAACTATGTCCATGCATATGTTAGACTCCAA

TTTTAAATTAAATTATTACCATCATTATTATTTCATTTTGCTTTCTAAGTTATAAAAACTCAAATATTCA

CCCAAAAAGTTTGGAATAGAATTCTATGCAATTCGTCTTAAATTGTATTGGTGACAATCAACAAATTATA

ATTAATATTGTTCTTACAATTGAATGGTTGAAATTTTTTTTATAGTTTTGGTAAATGGAAATTTTATAGA

GTTGTAGTTGAATAATGTCACTATCACTAATCGATGCAGTATTATACTTTGTTGACTTTTTTATTTTTAT

TTTTATTTTATTTTATAATTTACTCTCGTTTTACTGATATGTTTTTTTCAAAATTTTGGATAACTAACAA

AATTTATTTGTAATATTTATCAACAATGATTATATTATTAGCTCCATTGTTGTAAATTGTGATGACATAC

TATATCATTGTTTTTGCAGGTTTGACAGAAAACTGAAGAACACATACGACCACCTTCTCTTTAGCAGGTC

TTCCTCAATCCTAACACTTTTTGAAACATTTGTGATGTATGGAAGGGATAGAAATGATTGTCAATTACAA

GTGACTGCATCTTGTTGATTGCCAAGTTTAGATGGAAATTTTTATTGTGATTGCAAGTGATACCATTTTG

TTATTTAATAAAGGGTTTTCAATTTCCAGTACGTTGCAAGTGTCTGAGGATTTTAGGGATGAAGATCACT

TCATTTTTTGCTGTGTGACCTGAACTTATTTCTTCCTTTCTTTATCGATGGCATTTGTGGATTATAAATA

AAGCATTTTCATCACCTCACAACCTGTATATTTTTCTTTATGCAGAAGTCCCCTTTTGAGTGTATATGCT

GACATGTCAGTAACTTGCAAGGTAAAAGTCACTTCTTGCATGTCATTGATTTGCACTGATGTATTTCCAA

TTCCTGTATTTTTTGTTGTTTGACACTGAATTTCTTCTGCATACGTTTGTCTGGTTGATATATCATTAAA

TGTTGGAATATTGTAAGTAAGGGTAACTAACTCTGTTTCACTATCTTATGCAGGAGTATTATAACCCAAA

CCAATCTATGTTAGAGTTGGTTTTTGCACCAGCCGAAGAATGGATTTCACGTAGCGATGAAGATATTATT

CAAGCCACAATGTCTGAGCTTGCCAAACTCTTTCCTAATGAAATTTCCGCAGACCAGAGCAAAGCAAAGA

TTCTCAAGTACCACGTTGTTAAAACACCAAGGTTTGATTGAATGTTGGAATATTTCTTTTAGCTTCTTTT

GTAAACATGGATTGTGGAATGTCTATTTTGTTACTGAACTAATGACATTCATGGTCACTACCTTAAGTTG

TCTTGTGAATCCTAATGGAACTGATTACATTGTTTCAGGTCGGTTTACAAAACTGTTCCAAATTGTGAAC

CTTGTCGTCCCTTACAAAGATCTCCTATAGAGGGTTTCTATTTAGCTGGAGATTACACAAAACAAAAATA

TTTAGCTTCAATGGAAGGTGCCGTTCTTTCAGGGAAGTTTTGTGCACAGGCTATTGTACAGGTAAAATCT

GTCACAAAACTATCTATGTAACTCTCAGCAAATCATTAGCAAGAAATGCAATTGTCTATACATGTTTTCT

TAGTGATTTTGTGAGAGAAACTTCTTAATTATCAATACAGTAGGATGGATAACGCTATTTTGTGTTGTGG

AAATTCCATACTAGAGTTCACAGCTTTTATTTGTTAATTCAAAGAGTCTTTTCATTTATTATTTTGTGGT

TGAAACTTGAAACATTGTTATTGAGTTCCAATGCTCCTCATGCAAACAGGTTATGAACAATATTTGAAAT

TTATATGGAAGAGTTGGTGTAAATAGGATTGATCTTCTGTATAGGTTGATTTGAATGTTTTTCCTATCAC

TATATATCACATTGCTGTCATTGAGGCTTGCTAAAATGTTATGCAGGATTCTGAGCTACTTACTGCTCGG

GGCCAGAAAAGAATTGCTAAAGCGAGTGCTGTTTAACAATTATGGTACAGTACAGGAGCAACATTTCAAC

CTTTGCTTTTGGTATCTGTGATCAGGAGACAGTCAACTATATCAATTCATACGAAGAAAGAAAGGTTCAG

ACTCTGAAATTCAGCTAGACAGCGATAATATGTGCACTAAGCTACAGGGAACAAATGACATGTGTTAGTC

AATATTATAGTTTGGGTCCACCTGATAAGTGATTTTGTATTGGAACAGAATGTGTCATTCATGTGCCAAA

ATTGCAAGTGTACTGTTATCGTAAAATAATAAATTATTGTTCTCTCCACATGGACAGTGGTTTCTTTTTG

TTCCTTCTATGGGTTTTTCTTAGGCCAATGACTTATACACTCATTCCGCTCCCAATACTATAATGACTTA

TACATTGA

**PREDICTED: Cajanus cajan 15-cis-phytoene desaturase, chloroplastic/chromoplastic (LOC109797685), mRNA**

NCBI Reference Sequence: XM_020357799.2 >XM_020357799.2 PREDICTED: Cajanus cajan 15-cis-phytoene desaturase, chloroplastic/chromoplastic (LOC109797685), mRNA (size: 2233 bp)

ATTTTAAGAATTGTTGTCGTGATCTTCTTCATCATCCACCAGAGAATATTCAAACACACCGCACCAGACACCACTCCTCTCCTCTGCTGCTTTCTCCACCAAACTCACTTTCTCTCACTCTCAATCCAATGGCCGCATGTGGCTGCATATCTGTCTCCAACTTCAATTGCCTCCTTGGCGCCAGAAAACTATCCAAATTCGCTTCTTCAGATGCCACACTTTCCCTATCATTCGCTGCGAGCGACTCCATGGGTCTTACTCTGCGACCCACTTCAACTCTTTCTCCTAAGAGAAACTTTTTCTCTCCCTTGCGCGTCGTTTGCGCCGATTATCCTCGCCCGGAGCTCGAAAACACCGCCAATTTCATCGAAGCTGCTTACTTATCTTCCACGTTTCGTGCTTCTCCGCGGCCACAGAAACCCTTGAAGGTCGTTATTGCCGGTGCAGGATTGGCTGGTTTATCAACTGCAAAGTATTTGGCTGATGCTGGGCATACGCCTATATTGCTGGAAGCAAGAGACGTTCTAGGTGGAAAGGTTGCTGCTTGGAAAGACAAGGATGGAGACTGGTATGAGACTGGCCTGCACATCTTCTTTGGGGCTTACCCTAATGTGCAGAACCTATTTGGTGAACTTGGCATTAATGATCGGTTACAATGGAAGGAGCATTCCATGATTTTTGCTATGCCAAATAAGCCTGGAGAGTTTAGTCGATTTGATTTTCCTGATGCCTTTCCCGCCCCATTAAATGGAATATGGGCAATATTGAGGAACAATGAGATGCTGACATGGCCAGAGAAAGTCAAATTTGCAATTGGGCTTCTGCCAGCTATGCTTGGTGGACAACCATATGTTGAGGCTCAAGATGGTCTTTCTGTTAAGGAATGGATGGCAAGACAGGGAGTACCTGAACGAGTGACTGAAGAGGTGTTCATAGCAATGTCAAAGGCACTAAACTTTATCAATCCTGATGAACTTTCAATGCAATGTATATTAATTGCTTTAAACCGATTTCTTCAGGAGAAACATGGTTCTAAAATGGCCTTTTTGGACGGCAATCCCCCTGAAAGACTTTGTATGCCAATAGTTGATCATATTCAGTCCTTGGGTGGTGAAGTTCATCTCAATTCGCGCATTCAAAAAATTGAGCTCAATAATGATGGCACCGTGAAGAGCTTCTTACTAAACAATGGGAAGGTGATGGAAGGTGATGCTTATGTGTTTGCAACTCCAGTGGATATTCTGAAGCTTCTTCTACCTGACAACTGGAAAGGGATTCCATATTTCCAGAGATTGGATAAATTAGTTGGAGTCCCAGTCATTAATGTTCACATATGGTTTGACAGAAAACTGAAGAACACATACGACCACCTTCTCTTTAGCAGAAGTCCCCTTTTGAGTGTATATGCTGACATGTCAGTAACTTGCAAGGAGTATTATAACCCAAACCAATCTATGTTAGAGTTGGTTTTTGCACCAGCCGAAGAATGGATTTCACGTAGCGATGAAGATATTATTCAAGCCACAATGTCTGAGCTTGCCAAACTCTTTCCTAATGAAATTTCCGCAGACCAGAGCAAAGCAAAGATTCTCAAGTACCACGTTGTTAAAACACCAAGGTCGGTTTACAAAACTGTTCCAAATTGTGAACCTTGTCGTCCCTTACAAAGATCTCCTATAGAGGGTTTCTATTTAGCTGGAGATTACACAAAACAAAAATATTTAGCTTCAATGGAAGGTGCCGTTCTTTCAGGGAAGTTTTGTGCACAGGCTATTGTACAGGATTCTGAGCTACTTACTGCTCGGGGCCAGAAAAGAATTGCTAAAGCGAGTGCTGTTTAACAATTATGGTACAGTACAGGAGCAACATTTCAACCTTTGCTTTTGGTATCTGTGATCAGGAGACAGTCAACTATATCAATTCATACGAAGAAAGAAAGGTTCAGACTCTGAAATTCAGCTAGACAGCGATAATATGTGCACTAAGCTACAGGGAACAAATGACATGTGTTAGTCAATATTATAGTTTGGGTCCACCTGATAAGTGATTTTGTATTGGAACAGAATGTGTCATTCATGTGCCAAAATTGCAAGTGTACTG

TTATCGTAAAATAATAAATTATTGTTCTCTCCACATGGACAGTGGTTTCTTTTTGTTCCTTCTATGGGTTTTTCTTAGGCCAATGACTTATACACTCATTCCGCTCCCAATACTATAATGACTTATACATTGA

**>lcl|XM_020357799.2_cds_XP_020213388.1_1 [gene=LOC109797685] [db_xref=GeneID:109797685] [protein=15-cis-phytoene desaturase, chloroplastic/chromoplastic] [protein_id=XP_020213388.1] [location=129..1841] [gbkey=CDS](size: 1713 bp)**

ATGGCCGCATGTGGCTGCATATCTGTCTCCAACTTCAATTGCCTCCTTGGCGCCAGAAAACTATCCAAAT

TCGCTTCTTCAGATGCCACACTTTCCCTATCATTCGCTGCGAGCGACTCCATGGGTCTTACTCTGCGACC

CACTTCAACTCTTTCTCCTAAGAGAAACTTTTTCTCTCCCTTGCGCGTCGTTTGCGCCGATTATCCTCGC

CCGGAGCTCGAAAACACCGCCAATTTCATCGAAGCTGCTTACTTATCTTCCACGTTTCGTGCTTCTCCGC

GGCCACAGAAACCCTTGAAGGTCGTTATTGCCGGTGCAGGATTGGCTGGTTTATCAACTGCAAAGTATTT

GGCTGATGCTGGGCATACGCCTATATTGCTGGAAGCAAGAGACGTTCTAGGTGGAAAGGTTGCTGCTTGG

AAAGACAAGGATGGAGACTGGTATGAGACTGGCCTGCACATCTTCTTTGGGGCTTACCCTAATGTGCAGA

ACCTATTTGGTGAACTTGGCATTAATGATCGGTTACAATGGAAGGAGCATTCCATGATTTTTGCTATGCC

AAATAAGCCTGGAGAGTTTAGTCGATTTGATTTTCCTGATGCCTTTCCCGCCCCATTAAATGGAATATGG

GCAATATTGAGGAACAATGAGATGCTGACATGGCCAGAGAAAGTCAAATTTGCAATTGGGCTTCTGCCAG

CTATGCTTGGTGGACAACCATATGTTGAGGCTCAAGATGGTCTTTCTGTTAAGGAATGGATGGCAAGACA

GGGAGTACCTGAACGAGTGACTGAAGAGGTGTTCATAGCAATGTCAAAGGCACTAAACTTTATCAATCCT

GATGAACTTTCAATGCAATGTATATTAATTGCTTTAAACCGATTTCTTCAGGAGAAACATGGTTCTAAAA

TGGCCTTTTTGGACGGCAATCCCCCTGAAAGACTTTGTATGCCAATAGTTGATCATATTCAGTCCTTGGG

TGGTGAAGTTCATCTCAATTCGCGCATTCAAAAAATTGAGCTCAATAATGATGGCACCGTGAAGAGCTTC

TTACTAAACAATGGGAAGGTGATGGAAGGTGATGCTTATGTGTTTGCAACTCCAGTGGATATTCTGAAGC

TTCTTCTACCTGACAACTGGAAAGGGATTCCATATTTCCAGAGATTGGATAAATTAGTTGGAGTCCCAGT

CATTAATGTTCACATATGGTTTGACAGAAAACTGAAGAACACATACGACCACCTTCTCTTTAGCAGAAGT

CCCCTTTTGAGTGTATATGCTGACATGTCAGTAACTTGCAAGGAGTATTATAACCCAAACCAATCTATGT

TAGAGTTGGTTTTTGCACCAGCCGAAGAATGGATTTCACGTAGCGATGAAGATATTATTCAAGCCACAAT

GTCTGAGCTTGCCAAACTCTTTCCTAATGAAATTTCCGCAGACCAGAGCAAAGCAAAGATTCTCAAGTAC

CACGTTGTTAAAACACCAAGGTCGGTTTACAAAACTGTTCCAAATTGTGAACCTTGTCGTCCCTTACAAA

GATCTCCTATAGAGGGTTTCTATTTAGCTGGAGATTACACAAAACAAAAATATTTAGCTTCAATGGAAGG

TGCCGTTCTTTCAGGGAAGTTTTGTGCACAGGCTATTGTACAGGATTCTGAGCTACTTACTGCTCGGGGC

CAGAAAAGAATTGCTAAAGCGAGTGCTGTTTAA

**15-cis-phytoene desaturase, chloroplastic/chromoplastic [Cajanus cajan]**

NCBI Reference Sequence: XP_020213388.1 >XP_020213388.1 15-cis-phytoene desaturase, chloroplastic/chromoplastic [Cajanus cajan] (size: 570 bp)

MAACGCISVSNFNCLLGARKLSKFASSDATLSLSFAASDSMGLTLRPTSTLSPKRNFFSPLRVVCADYPRPELENTANFIEAAYLSSTFRASPRPQKPLKVVIAGAGLAGLSTAKYLADAGHTPILLEARDVLGGKVAAWKDKDGDWYETGLHIFFGAYPNVQNLFGELGINDRLQWKEHSMIFAMPNKPGEFSRFDFPDAFPAPLNGIWAILRNNEMLTWPEKVKFAIGLLPAMLGGQPYVEAQDGLSVKEWMARQGVPERVTEEVFIAMSKALNFINPDELSMQCILIALNRFLQEKHGSKMAFLDGNPPERLCMPIVDHIQSLGGEVHLNSRIQKIELNNDGTVKSFLLNNGKVMEGDAYVFATPVDILKLLLPDNWKGIPYFQRLDKLVGVPVINVHIWFDRKLKNTYDHLLFSRSPLLSVYADMSVTCKEYYNPNQSMLELVFAPAEEWISRSDEDIIQATMSELAKLFPNEISADQSKAKILKYHVVKTPRSVYKTVPNCEPCRPLQRSPIEGFYLAGDYTKQKYLASMEGAVLSGKFCAQAIVQDSELLTARGQKRIAKASAV

**Supplementary file 6 C:** Represents the CcPDS gene sgRNA cassette (single sgRNA)

**PDS gene conserved sgRNA cassette (single sgRNA)**

5’CTCGCGAGACCTTTACTTTAAATTTTTTCTTATGCAGCCTGTGATGGATAACTGAATCAAACAAATGGCGTCTGGGTTTAAGAAGATCTGTTTTGGCTATGTTGGACGAAACAAGTGAACTTTTAGGATCAACTTCAGTTTATATATGGAGCTTATATCGAGCAATAAGATAAGTGGGCTTTTTATGTAATTTAATGGGCTATCGTCCATAGATTCACTAATACCCATGCCCAGTACCCATGTATGCGTTTCATATAAGCTCCTAATTTCTCCCACATCGCTCAAATCTAAACAAATCTTGTTGTATATATAACACTGAGGGAGCAACATTGGTCA**GATCATATTCAGTCCTTGGG**GTTTTAGAGCTAGAAATAGCAAGTTAAAATAAGGCTAGTCCGTTATCAACTTGAAAAAGTGGCACCGAGTCGGTGCTTTTTTTTTCTAGACCCAGCTTTCTTGTACAAAGTTGGCATTAGGTCTCGCGGT3’

AtU3b promoter

White sgRNA: GATCATATTCAGTCCTTGGG

Scaffold: GTTTTAGAGCTAGAAATAGCAAGTTAAAATAAGGCTAGTCCGTTATCAACTTGAAAAAGTGGCACCGAGTCGGTGCT

Sequence for terminal portion of gRNA: Poly T + Linker TTTTTTTTCTAGACCCAGCTTTCTTGTACAAAGTTGGCATTAGGTCTCGCGGT

GGTCTC: BsaI site

**Gene specific primer of Cc_PDS: Selected gene sequence**

**>Cc_pds**

ATTTTTTTAGCACAATAAGAAAAAGAAATGAATAAGGAAAATTGGTTTTAAAAAAGGGAAGATAAAGAAG

AAATAATTTCTTTCTCTTCCCCCATTCATATAAGGAGGGTTGTCTATGTCTGCATGCCTTTATTAGCATA

GTTGATTCATTTGCTATTATTGAAAGAAAAATCAAACATGGGAGACAAACTGGGATATAGTTGAAGTTGT

TTGTTTAGGTTTGGCTTGTACCATTATTGAAATACTAGTCATTGTGATCTGGATTGATGTGAAGAAGGGG

CTTCTGTGCTCTAAGAATGGGAGAATAAATTAATTTTGTATATTAGATTTTGCTTTGTCTCTTAATTTAA

TGATGACAGTTGAGTATTTTACTTTACTGGAAAATATAGAAAGGATATCATAGTTGTAGTTGTTAGATTG

TATTACTTGATATTTACGTAATGCTTTAAAATATATTTCATCAGGACTGAATGCAACCCTCTCATGGTCT

GTATTGCAAATAATTTGAGAATAACAAAATGTTTTCTCCTTATTGTAGGGAGTACCTGAACGAGTGACTG

AAGAGGTGTTCATAGCAATGTCAAAGGCACTAAACTTTATCAATCCTGATGAACTTTCAATGCAATGTAT

ATTAATTGCTTTAAACCGATTTCTTCAGGTGTGATCATTTCCTTTCATTAAACACTATTCCAATGGCATT

ATTGTGTGATTTTTTAGGTCATTTCAATCTCATTATAACAGTTGAGTGGAATCAGATTAGTAAGCTCTAA

GATGGCCTTTTATTTCACAAACATGTTTTGTGGAACTTGGGATCAATTTTTTCCTTCAACTAATTGTGAT

ATTGTTAAATTCTTGAATAGGAGAAACATGGTTCTAAAATGGCCTTTTTGGACGGCAATCCCCCTGAAAG

ACTTTGTATGCCAATAGTTGATCATATTCAGTCCTTGGGTGGTGAAGTTCATCTCAATTCGCGCATTCAA

AAAATTGAGCTCAATAATGATGGCACCGTGAAGAGCTTCTTACTAAACAATGGGAAGGTGATGGAAGGTG

ATGCTTATGTGTTTGCAACTCCAGGTATTTTTTTTTCTTATTTTCCCACTGTGTTAATTAATTGTTTTCT

TGGCATCTTAATCTCCATAACATTTTTGAGGAATTTCCACCCTTGAAAACAGTGGATATTCTGAAGCTTC

TTCTACCTGACAACTGGAAAGGGATTCCATATTTCCAGAGATTGGATAAATTAGTTGGAGTCCCAGTCAT

TAATGTTCACATATGGTAAGTAACTGCTTTTGATGTTGGAAACTATGTCCATGCATATGTTAGACTCCAA

TTTTAAATTAAATTATTACCATCATTATTATTTCATTTTGCTTTCTAAGTTATAAAAACTCAAATATTCA

CCCAAAAAGTTTGGAATAGAATTCTATGCAATTCGTCTTAAATTGTATTGGTGACAATCAACAAATTATA

ATTAATATTGTTCTTACAATTGAATGGTTGAAATTTTTTTTATAGTTTTGGTAAATGGAAATTTTATAGA

GTTGTAGTTGAATAATGTCACTATCACTAATCGATGCAGTATTATACTTTGTTGACTTTTTTATTTTTAT

TTTTATTTTATTTTATAATTTACTCTCGTTTTACTGATATGTTTTTTTCAAAATTTTGGATAACTAACAA

AATTTATTTGTAATATTTATCAACAATGATTATATTATTAGCTCCATTGTTGTAAATTGTGATGACATAC

TATATCATTGTTTTTGCAGGTTTGACAGAAAACTGAAGAACACATACGACCACCTTCTCTTTAGCAGGTC

TTCCTCAATCCTAACACTTTTTGAAACATTTGTGATGTATGGAAGGGATAGAAATGATTGTCAATTACAA

GTGACTGCATCTTGTTGATTGCCAAGTTTAGATGGAAATTTTTATTGTGATTGCAAGTGATACCATTTTG

TTATTTAATAAAGGGTTTTCAATTTCCAGTACGTTGCAAGTGTCTGAGGATTTTAGGGATGAAGATCACT

TCATTTTTTGCTGTGTGACCTGAACTTATTTCTTCCTTTCTTTATCGATGGCATTTGTGGATTATAAATA

AAGCATTTTCATCACCTCACAACCTGTATATTTTTCTTTATGCAGAAGTCCCCTTTTGAGTGTATATGCT

GACATGTCAGTAACTTGCAAGGTAAAAGTCACTTCTTGCATGTCATTGATTTGCACTGATGTATTTCCAA

TTCCTGTATTTTTTGTTGTTTGACACTGAATTTCTTCTGCATACGTTTGTCTGGTTGATATATCATTAAA

**Supplementary file 6 D: Represents the *CcGAPDH* nucleotide sequence**

**PREDICTED: Cajanus cajan glyceraldehyde-3-phosphate dehydrogenase, cytosolic (LOC109796067), mRNA**

NCBI Reference Sequence: XM_020355692.2

[GenBank](https://www.ncbi.nlm.nih.gov/nuccore/XM_020355692.2?report=genbank) [Graphics](https://www.ncbi.nlm.nih.gov/nuccore/XM_020355692.2?report=graph)

>XM_020355692.2 PREDICTED: Cajanus cajan glyceraldehyde-3-phosphate dehydrogenase, cytosolic (LOC109796067), mRNA

AGGTGTCACGCACTTCACACATATATAATTATAATAATTGCTCTCTTTTATACCTCCATAAAACCCTATC

TCACACTTCAATTCTCAACTTCTTCTACCCACTCTAACACCAACCGCTCTATTCTCCGCAGTCATGGGCA

AGGTCAAGATCGGAATCAACGGATTCGGAAGAATTGGCCGTTTGGTGGCCAGGGTTGCTCTTCAGAGAGA

CGATGTGGAACTCGTTGCCGTTAACGATCCCTTCATTACCACCGATTACATGACGTACATGTTTAAATAC

GACAGTGTTCACGGACACTGGAAGCATCACGAAGTTTCCGTCAAGGACTCCAAGACCCTTCTCTTTGGTG

ACAAGCCAGTCACTGTTTTTGGTCACAGGAACCCTGAAGAGATCCCGTGGGCCGAGACTGGAGCTGAAAT

CATTGTTGAGTCCACCGGAGTTTTCACCGATAAGGACAAGGCCGCTGCACATTTGAAGGGTGGTGCGAAG

AAGGTTATTATCTCTGCTCCCAGCAAGGATGCTCCCATGTTTGTTGTTGGTGTTAACGAACATGAGTACA

AGCCAGAGCTTGATATTATTTCCAATGCTAGCTGCACAACTAACTGCCTTGCTCCACTCGCCAAGGTTAT

TAATGACAGGTTTGGCATTGTTGAGGGTTTGATGACCACTGTTCATTCCATCACTGCTACTCAGAAGACT

GTCGATGGGCCATCAGCCAAAGACTGGAGAGGTGGAAGGGCTGCTTCATTTAACATCATTCCTAGCAGCA

CCGGAGCTGCCAAGGCTGTTGGGAAAGTCCTCCCATCTTTGAATGGAAAATTGACTGGAATGGCATTCCG

TGTTCCTACTGTCGATGTCTCTGTTGTTGACCTCACAGTGAGGTTGGAGAAGCCAGCTTCATATGAAGAT

ATTAAAAATGCTATCAAGGAGGAGTCAGAGGGCAAGTTGAAGGGAATTCTTGGTTACACTGAAGATGATG

TGGTCTCCAGCGACTTTGTGGGTGATAGCAGATCAAGCATTTTTGATGCAAAGGCTGGAATTGCATTGAG

TAAGAATTTTGTGAAGCTTGTGTCGTGGTACGACAATGAGTGGGGATACAGCTCCCGCGTGATTGACCTG

CTTGTTCACGTTGCCAAGAAGTCTGCATAAATTGTAACTTATGTTTATGTCTAGCTGCGATTTAGTGTCT

TGCTCGTGCAAAAATGAGAATTCTGAATAAATCGGTTTCTGGTACCAGTGGTGTTACCCTGTTGGGAGCA

TTAGCTCTTTTTGGACTTTTGGTGTTTTCCTCTTTTGTGGAGGAGATGGAGTTTTTGGATTTTCATAAAC

TTATTGGATGTACGTGGCTTATATACTTTGATCGTTATAATAGTTATTTGGCCGTCCTCCATTTTCAA

**PREDICTED: Cajanus cajan glyceraldehyde-3-phosphate dehydrogenase, cytosolic (LOC109796067), mRNA**

NCBI Reference Sequence: XM_020355692.2

[GenBank](https://www.ncbi.nlm.nih.gov/nuccore/XM_020355692.2?report=genbank) [Graphics](https://www.ncbi.nlm.nih.gov/nuccore/XM_020355692.2?report=graph)

>XM_020355692.2:134-1150 PREDICTED: Cajanus cajan glyceraldehyde-3-phosphate dehydrogenase, cytosolic (LOC109796067), mRNA

ATGGGCAAGGTCAAGATCGGAATCAACGGATTCGGAAGAATTGGCCGTTTGGTGGCCAGGGTTGCTCTTC

AGAGAGACGATGTGGAACTCGTTGCCGTTAACGATCCCTTCATTACCACCGATTACATGACGTACATGTT

TAAATACGACAGTGTTCACGGACACTGGAAGCATCACGAAGTTTCCGTCAAGGACTCCAAGACCCTTCTC

TTTGGTGACAAGCCAGTCACTGTTTTTGGTCACAGGAACCCTGAAGAGATCCCGTGGGCCGAGACTGGAG

CTGAAATCATTGTTGAGTCCACCGGAGTTTTCACCGATAAGGACAAGGCCGCTGCACATTTGAAGGGTGG

TGCGAAGAAGGTTATTATCTCTGCTCCCAGCAAGGATGCTCCCATGTTTGTTGTTGGTGTTAACGAACAT

GAGTACAAGCCAGAGCTTGATATTATTTCCAATGCTAGCTGCACAACTAACTGCCTTGCTCCACTCGCCA

AGGTTATTAATGACAGGTTTGGCATTGTTGAGGGTTTGATGACCACTGTTCATTCCATCACTGCTACTCA

GAAGACTGTCGATGGGCCATCAGCCAAAGACTGGAGAGGTGGAAGGGCTGCTTCATTTAACATCATTCCT

AGCAGCACCGGAGCTGCCAAGGCTGTTGGGAAAGTCCTCCCATCTTTGAATGGAAAATTGACTGGAATGG

CATTCCGTGTTCCTACTGTCGATGTCTCTGTTGTTGACCTCACAGTGAGGTTGGAGAAGCCAGCTTCATA

TGAAGATATTAAAAATGCTATCAAGGAGGAGTCAGAGGGCAAGTTGAAGGGAATTCTTGGTTACACTGAA

GATGATGTGGTCTCCAGCGACTTTGTGGGTGATAGCAGATCAAGCATTTTTGATGCAAAGGCTGGAATTG

CATTGAGTAAGAATTTTGTGAAGCTTGTGTCGTGGTACGACAATGAGTGGGGATACAGCTCCCGCGTGAT

TGACCTGCTTGTTCACGTTGCCAAGAAGTCTGCATAA
