# Supplementary material for: From recalcitrance to precision: a robust regeneration, transformation and targeted gene editing framework in Cajanus cajan
Source: Front Genome Ed. 2026 Jun 9;8:1815812. doi: 10.3389/fgeed.2026.1815812 (PMC13287048; doi:10.3389/fgeed.2026.1815812)
Supplement: Supplementary file 5 [file Supplementaryfile5.docx]

**Supplementary file 5 A: Figure S1**. The NICTK-2_pCRISPR-Cas9 plant transformation vector, which is 14.5 kb in size, includes a 345 bp CaMV 35S promoter and a 175 bp CaMV Poly(A) signal terminator. Furthermore, this vector included a multiple cloning site (MCS) with four restriction sites—*BsaI-BsaI, SwaI-SbfI, SwaI-AsiSI, and SbfI-AsiSI*.


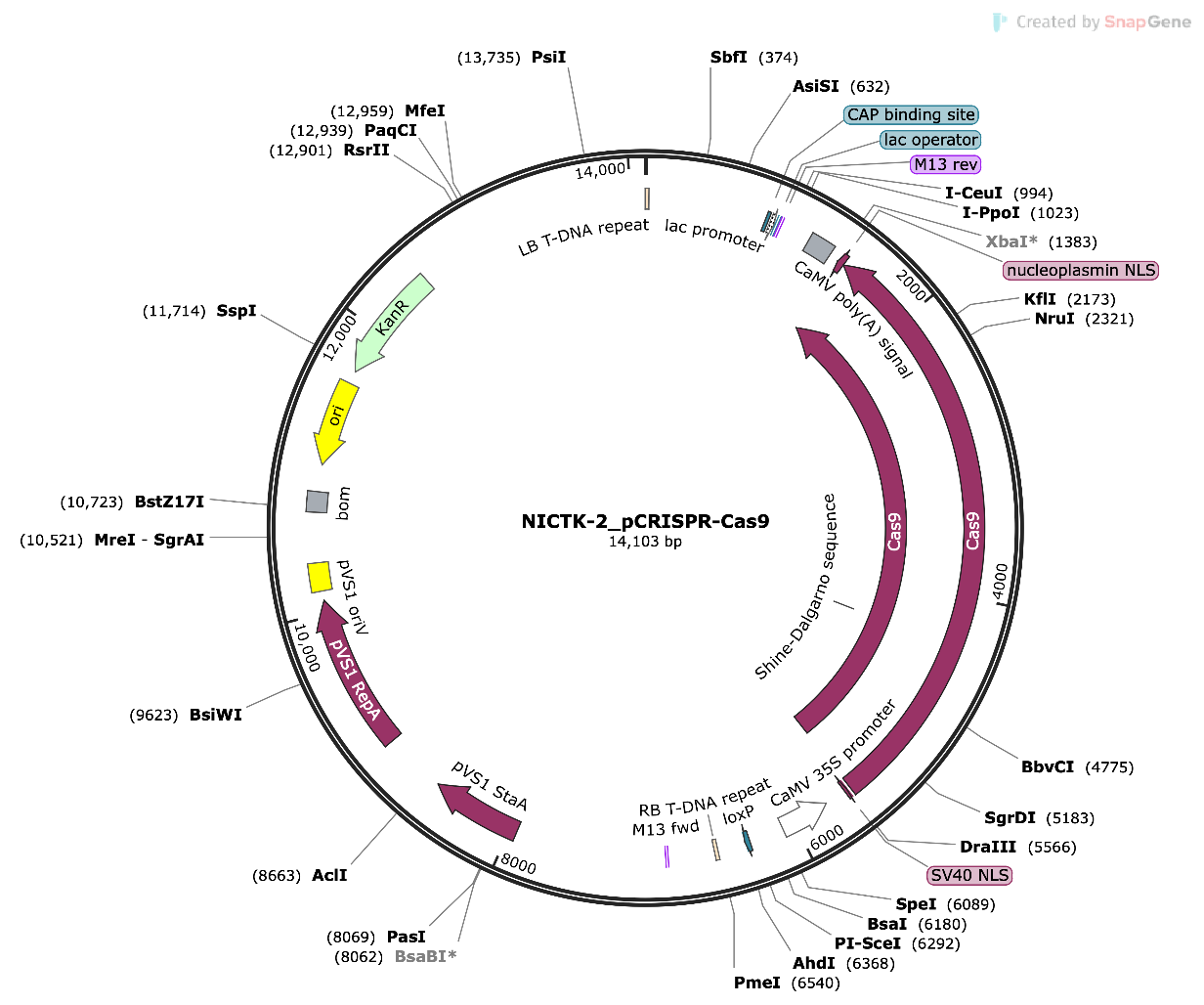


**Supplementary file 5 B: Sequence. NICTK-2_pCRISPR-Cas9 vector sequence**

TGGCAGGATATATTGTGGTGTAAACAAATTGACGCTTAGACAACTTAATAACACATTGCGGACGTTTTTAATGTACTGAATTAACGCCGAATTAATTCGGGG**GCT**ATGA**ATTTAAAT**GAAACAGCTAGCGTCAAGCTATTTGAGCGCTAAGTCCTATGTAGTATCGCGTCAAGCTATTGTAAGGTCGATTAGTAGCCACTCAAGAGCTAGTGACCAAGCTTTAGCTGGCTAAGTCCTATTAAGTCCTAGTTCCAAGGCGCGTCAAGCATGCGGAGTTAACGATTAGTGACCAAATTTACTGCTCGTACTATGAGCATAGAATGCGCGTAGTGACCAAAATGTTTAAGTCCTACGTGCACTCAAGGCTTCCTGCAGGTAGTGCACTCAAGATTGATAAAAGTCAAGCCACTCAAGGCTAGTATGTAGCTAAGTCCTATTAAGTCAGGTCGCTAAGTTCAACAGCTATGTGCTCGTACTATGACCACTCAAGGCTGATAACCAGCTATGACCAAGCTAAGTCCTATAGTAGCCACTCAAGCCTATAGTTCAGTAGCCACTCAAGACAGCTATGTGCTTAAGTCATAGTTCAGTAAATGCTGAATGCATGGGCTGCAGTACTTGTTGCCT**GCGATCGC**GTATGC**TAT**ATAGCCCTTTGGTCTTCTGAGACTGTATCTTTGATATTCTTGGAGTAGACGAGAGTGTCGTGCTCCACCATGTTGGCAAGCTGCTCTAGCCAATACGCAAACCGCCTCTCCCCGCGCGTTGGCCGATTCATTAATGCAGCTGGCACGACAGGTTTCCCGACTGGAAAGCGGGCAGTGAGCGCAACGCAATTAATGTGAGTTAGCTCACTCATTAGGCACCCCAGGCTTTACACTTTATGCTTCCGGCTCGTATGTTGTGTGGAATTGTGAGCGGATAACAATTTCACACAGGAAACAGCTATGACATGATTACGAATTCGAGCTCGGTACCGTAACTATAACGGTCCTAAGGTAGCGAAGGATCCGCTCGCTACCTTAAGAGAGGATATCCCTCCATCCTATAATGTAGGCTATAGGAACTAGGGCAAGGCCGGCCATGCGGCCGCAAG**AAG**GCTGCAAG**GCCCGGGC**GAGAAGAGCGTAGTTGTTAGCGTTAGCGTTAGCGCAGTAGAGTTTGTAGATCTGGATTTTAGTACTGGATTTTGGTTTTAGGAATTAGAAATTTTATTGATAGAAGTATTTTACAAATACAAATACATACTAAGGGTTTCTTATATGCTCAACACATGAGCGAAACCCTATAGGAACCCTAATTCCCTTATCTGGGAACTACTCACACATTATTATGGAGAAAATAGAGAGAGATAGATTTGTAGAGAGAGACTGGTGATTTTTGCGGACTCTAGATCAGGTGGATCCTACTTCTTTTTCTTAGCCTGTCCGGCCTTTTTGGTGGCAGCAGGACGCTTATCACCACCAAGCTGGGAAAGGTCGATACGAGTCTCGTAAAGACCGGTGATGGACTGGTGGATGAGAGTAGCGTCGAGAACCTCCTTGGTGGACGTGTAACGCTTCCTGTCGATGGTGGTGTCGAAGTACTTGAAAGCAGCAGGGGCGCCGAGGTTCGTGAGCGTGAAGAGGTGGATGATGTTCTCGGCCTGCTCGCGGATGGGCTTGTCGCGGTGCTTGTTGTAGGCGGAGAGGACCTTGTCGAGGTTAGCGTCAGCGAGGATGACGCGCTTGGAGAACTCGGAGATCTGCTCGATGATCTCGTCGAGGTAGTGCTTGTGCTGCTCCACGAAGAGCTGCTTCTGCTCGTTATCCTCAGGGGAACCCTTGAGCTTCTCGTAGTGGGAGGCGAGGTAGAGGAAGTTCACGTACTTGGAAGGAAGAGCAAGCTCGTTACCCTTCTGGAGCTCACCAGCGGAAGCCAGCATCCTCTTACGACCGTTCTCGAGCTCGAAAAGAGAGTACTTGGGGAGCTTGATGATGAGGTCCTTCTTGACCTCCTTGTAGCCCTTGGCCTCGAGGAAGTCGATCGGGTTCTTCTCGAAGGAGGAGCGCTCCATGATGGTGATGCCGAGGAGCTCCTTGACGGACTTGAGCTTCTTCGACTTACCCTTCTCCACCTTGGCGACCACGAGGACGGAGTAGGCGACAGTAGGGGAGTCGAAACCACCGTACTTCTTAGGGTCCCAATCCTTCTTCCTAGCGATGAGCTTGTCCGAGTTTCTCTTTGGAAGGATAGACTCCTTGGAGAAGCCACCGGTCTGGACCTCGGTCTTCTTGACGATGTTAACCTGAGGCATAGAAAGGACCTTGCGAACAGTAGCGAAGTCGCGACCCTTGTCCCAAACGATCTCACCAGTCTCACCGTTCGTCTCGATAAGAGGGCGCTTGCGGATCTCGCCGTTGGCGAGGGTGATCTCGGTCTTGAAGAAGTTCATGATGTTGGAGTAGAAGAAGTACTTGGCGGTGGCCTTGCCGATCTCCTGCTCGGACTTGGCGATCATCTTACGAACGTCGTAGACCTTGTAGTCACCGTAGACGAACTCGGACTCAAGCTTAGGGTACTTCTTGATAAGAGCGGTACCAACGACAGCGTTAAGGTAAGCATCGTGAGCGTGGTGGTAGTTGTTGATCTCGCGGACCTTGTAGAACTGGAAGTCCTTGCGGAAGTCGGAGACGAGCTTGGACTTGAGGGTGATCACCTTGACCTCGCGGATGAGCTTGTCGTTCTCGTCGTACTTGGTGTTCATCCTAGAATCGAGGATCTGAGCAACGTGCTTGGTAATCTGCCTCGTCTCAACAAGCTGCCTCTTGATGAAACCAGCCTTGTCAAGCTCGGAAAGGCCACCCCTCTCAGCCTTCGTGAGGTTGTCGAACTTCCTCTGGGTAATGAGCTTAGCGTTGAGAAGCTGCCTCCAGTAGTTCTTCATCTTCTTGACAACCTCCTCGGAAGGGACGTTGTCCGACTTACCCCTGTTCTTGTCGGACCTCGTGAGGACCTTGTTGTCGATGGAGTCATCCTTAAGGAAAGACTGAGGAACAATGTGGTCGACGTCGTAGTCAGAAAGCCTGTTGATGTCGAGCTCCTGGTCAACGTACATATCCCTACCGTTCTGGAGGTAGTAGAGGTAGAGCTTCTCGTTCTGGAGCTGGGTGTTCTCGACAGGGTGCTCCTTAAGGATCTGAGAACCAAGCTCCTTGATACCCTCCTCAATCCTCTTCATGCGCTCCCTCGAGTTCTTCTGACCCTTCTGGGTAGTCTGGTTCTCACGAGCCATCTCGATGACGATGTTCTCAGGCTTGTGACGACCCATAACCTTGACAAGCTCATCGACAACCTTAACAGTCTGAAGGATGCCCTTCTTGATAGCAGGGGAACCAGCAAGGTTAGCAATGTGCTCGTGGAGAGAGTCGCCCTGACCGGACACCTGAGCCTTCTGAATATCCTCCTTGAAGGTAAGAGAGTCATCGTGGATGAGCTGCATGAAGTTCCTGTTAGCGAAACCATCAGACTTGAGGAAGTCGAGGATAGTCTTGCCGCTCTGCTTATCCCTGATACCGTTGATGAGCTTGCGGGAGAGCCTACCCCAACCGGTGTAACGGCGACGCTTGAGCTGCTTCATAACCTTGTCATCGAAGAGATGAGCGTAAGTCTTGAGCCTCTCCTCGATCATCTCCCTATCCTCGAAGAGAGTAAGAGTGAGGACGATGTCCTCGAGGATGTCCTCGTTCTCCTCGTTGTCGAGGAAGTCCTTGTCCTTGATGATCTTGAGGAGATCGTGGTAGGTACCGAGAGAAGCGTTGAAACGGTCCTCAACGCCGCTGATCTCGACGGAGTCGAAGCACTCGATCTTCTTGAAGTAGTCCTCCTTGAGCTGCTTGACGGTGACCTTGCGGTTGGTCTTGAAGAGGAGGTCAACGATAGCCTTCTTCTGCTCGCCGGAGAGGAAGGCAGGCTTGCGCATACCCTCGGTGACGTACTTGACCTTGGTGAGCTCGTTGTAGACCGTGAAGTACTCGTAGAGGAGGGAGTGCTTGGGGAGGACCTTCTCGTTGGGGAGGTTCTTGTCGAAGTTGGTCATGCGCTCGATGAAGGACTGGGCGGAAGCACCCTTGTCAACAACCTCCTCGAAGTTCCAAGGGGTGATAGTCTCCTCGGACTTCCTAGTCATCCAAGCGAAACGGGAGTTACCACGAGCAAGAGGACCAACGTAGTAAGGAATACGGAAAGTAAGGATCTTCTCGATCTTCTCACGGTTGTCCTTGAGGAAAGGGTAGAAGTCCTCCTGCCTACGAAGGATGGCGTGAAGCTCACCAAGGTGGATCTGGTGAGGGATAGAGCCGTTATCGAAAGTCCTCTGCTTCCTAAGGAGGTCCTCACGGTTAAGCTTAACGAGAAGCTCCTCAGTACCATCCATCTTCTCAAGGATTGGCTTGATGAACTTGTAGAACTCCTCCTGGCTAGCTCCACCGTCAATGTAACCGGCGTAGCCGTTCTTGGACTGGTCGAAGAAGATCTCCTTGTACTTCTCGGGGAGCTGCTGACGAACAAGAGCCTTGAGGAGGGTGAGGTCCTGGTGGTGCTCGTCGTAGCGCTTGATCATGGAAGCAGAAAGAGGAGCCTTAGTGATCTCAGTGTTAACCCTAAGGATATCCGAAAGGAGGATAGCATCGGAGAGGTTCTTAGCAGCAAGGAAGAGATCAGCGTACTGATCTCCAATCTGAGCGAGGAGGTTGTCGAGATCATCGTCGTAGGTATCCTTGGAGAGCTGAAGCTTAGCGTCCTCAGCGAGGTCGAAGTTGGACTTGAAGTTAGGGGTCAGACCGAGGGAGAGAGCGATGAGGTTACCGAAAAGACCGTTCTTCTTCTCACCAGGGAGCTGGGCGATGAGGTTCTCGAGACGCCTGGACTTGGAGAGCCTAGCGGAAAGGATCGCCTTAGCGTCGACACCGGAAGCGTTGATAGGGTTCTCCTCGAAGAGCTGGTTGTAGGTCTGGACGAGCTGGATGAAGAGCTTGTCCACGTCGGAGTTATCAGGGTTAAGGTCACCCTCGATAAGGAAGTGACCACGGAACTTGATCATGTGAGCGAGAGCAAGGTAGATGAGACGAAGATCAGCCTTATCAGTAGAGTCAACAAGCTTCTTACGAAGGTGGTAGATAGTGGGGTACTTCTCGTGGTAGGCGACCTCGTCGACGATGTTGCCGAAGATGGGGTGGCGCTCGTGCTTCTTGTCCTCCTCCACGAGGAAGGACTCCTCGAGGCGGTGGAAGAAGGAGTCGTCGACCTTCGCCATCTCGTTGGAGAAGATCTCCTGGAGGTAGCAGATGCGGTTCTTGCGGCGGGTGTAGCGGCGGCGGGCGGTGCGCTTGAGGCGGGTCGCCTCCGCCGTCTCGCCGGAGTCGAAGAGGAGGGCGCCGATGAGGTTCTTCTTGATGGAGTGGCGGTCGGTGTTGCCCAGGACCTTGAACTTCTTGGACGGGACCTTGTACTCGTCGGTGATCACCGCCCAGCCGACGCTGTTGGTGCCGATGTCGAGGCCGATGGAGTACTTCTTGTCAGCCGCAGGCACCCCGTGAATACCAACCTTCCGCTTCTTCTTAGGAGCCATGGCTATCGTTCGTAAATGGTGAAAATTTTCAGAAAATTGCTTTTGCTTTAAAAGAAATGATTTAAATTGCTGCAATAGAAGTAGAATGCTTGATTGCTTGAGATTCGTTTGTTTTGTATATGTTGTGTTGAGAACTCTCGACGTCCTCTCCAAATGAAATGAACTTCCTTATATAGAGGAAGGGTCTTGCGAAGGATAGTGGGATTGTGCGTCATCCCTTACGTCAGTGGAGATATCACATCAATCCACTTGCTTTGAAGACGTGGTTGGAACGTCTTCTTTTTCCACGATGCTCCTCGTGGGTGGGGGTCCATCTTTGGGACCACTGTCGGCAGAGGCATCTTCAACGATGGCCTTTCCTTTATCGCAATGATGGCATTTGTAGGAGCCACCTTCCTTTTCCACTATCTTCACAATAAAGTGACAGATAGCTGGGCAATGGAATCCGAGGAGGTTTCCGGATATTACCCTTTGTTGAAAAGTCTCACTAGTAATAAATGATTTAGTTTCAGAAAATTTAAAAGTCGTATGC **GCCCGGGC**
