# Supplementary material for: From recalcitrance to precision: a robust regeneration, transformation and targeted gene editing framework in Cajanus cajan
Source: Front Genome Ed. 2026 Jun 9;8:1815812. doi: 10.3389/fgeed.2026.1815812 (PMC13287048; doi:10.3389/fgeed.2026.1815812)
Supplement: Supplementary file 7 [file Supplementaryfile7.docx]

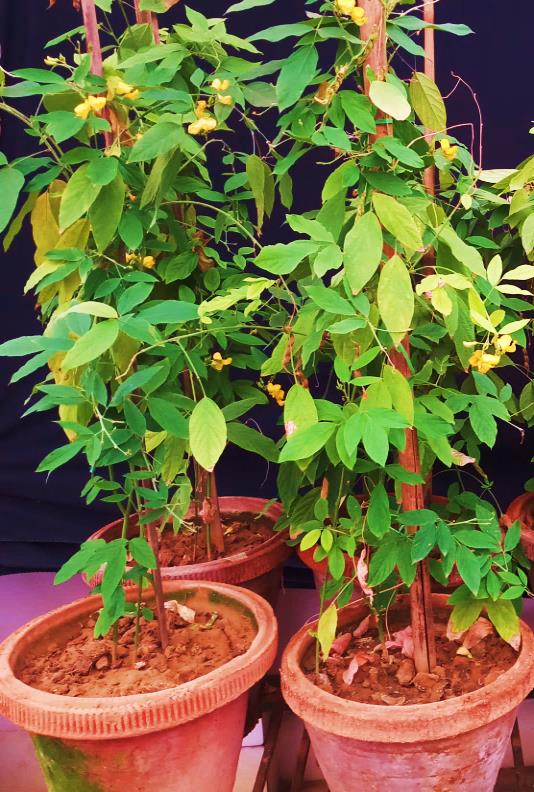

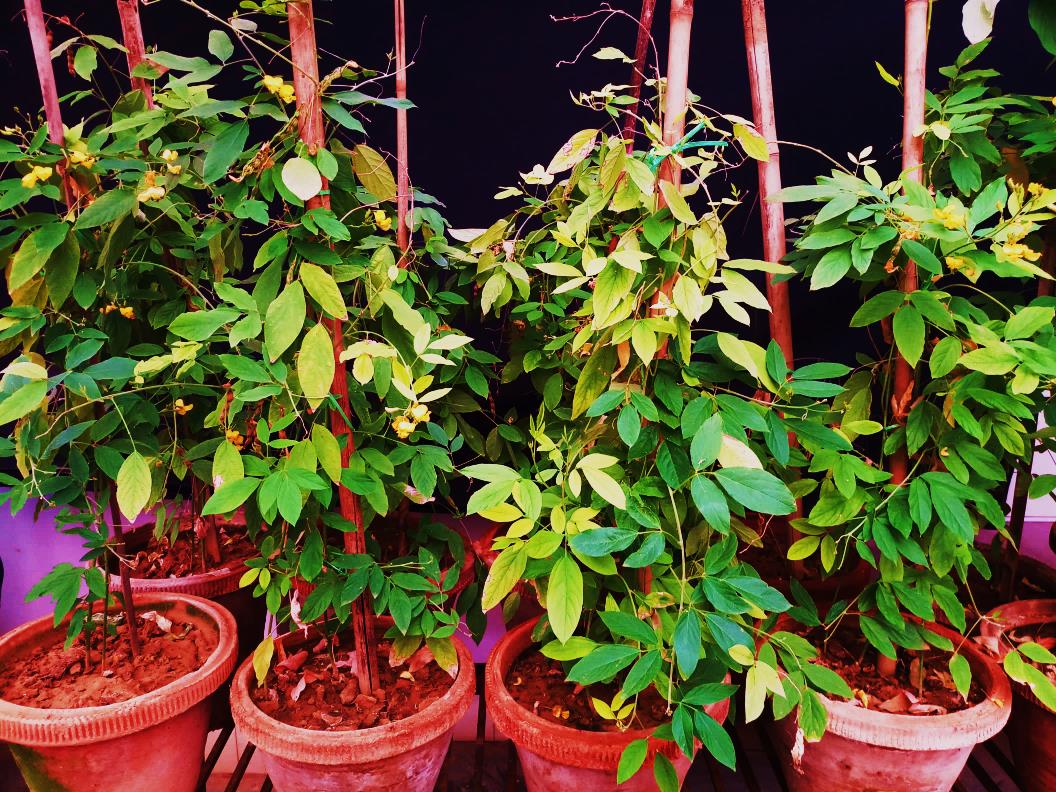


**A**

**B**

**Supplementary file 7: Figure S2.** Comparative morphological features of mature WT and T_1_ plants generated in-vitro in the glass house. They clearly displayed normal morphological features and growth habit when compared with WT. (A) wild type (WT) pigeonpea plants. (B) Regenerated transformed (NIC-TK2-pCRISPR/Cas9) plants**.**


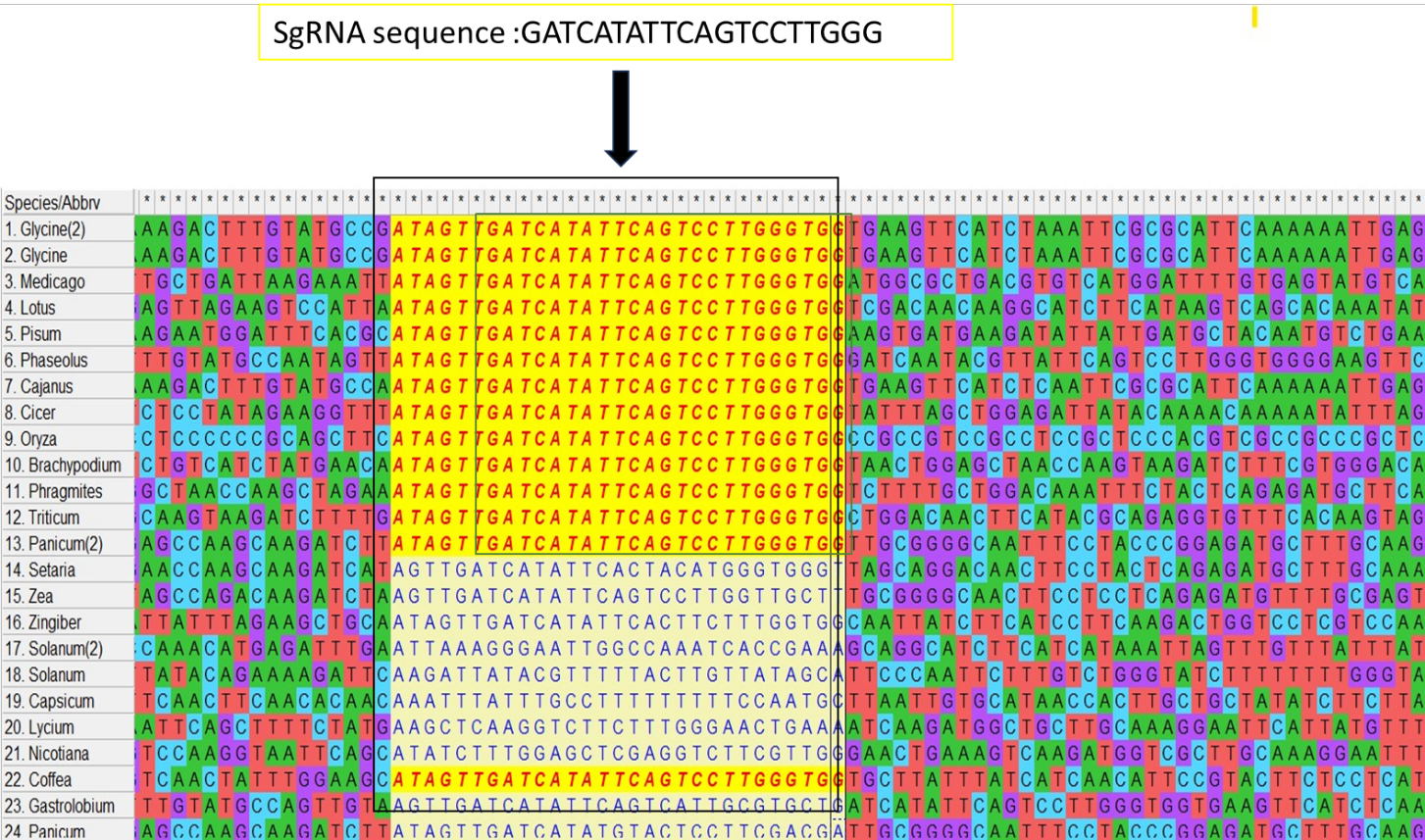


**Supplementary file 7: Figure S3.** Sequence alignment showed conserved sequences of PDS genes from the various crop, which were utilized for designing a single guide RNA for targeting the *Cc*PDS gene from pigeonpea.


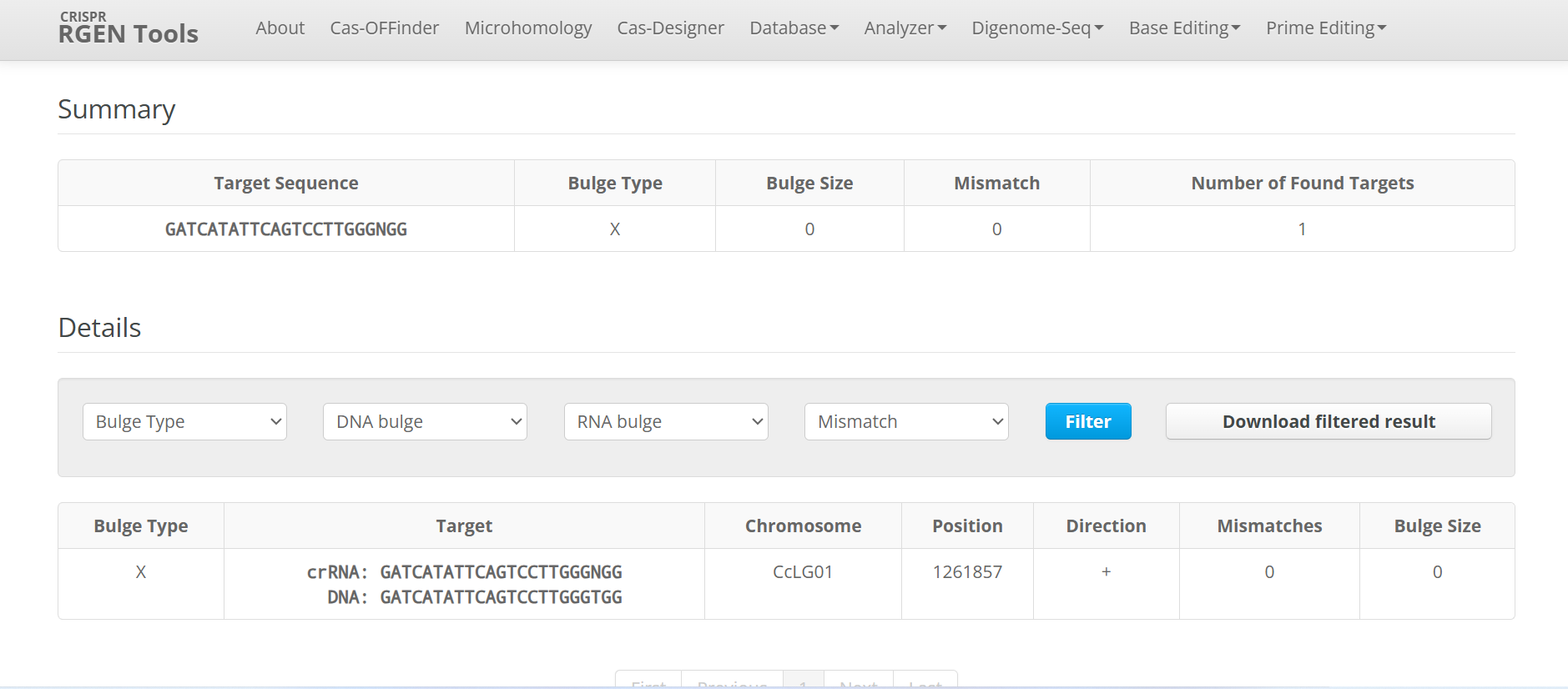


**Supplementary file S7: Figure S4.** The image shows the off-target analysis results for the PDS target specific selected sgRNAs by utilizing the Cas-OFFinder/CRISPR RGENE ([www.rgenome.net](http://www.rgenome.net/)) tool. The results show no off-target in the whole genome of the pigeonpea.


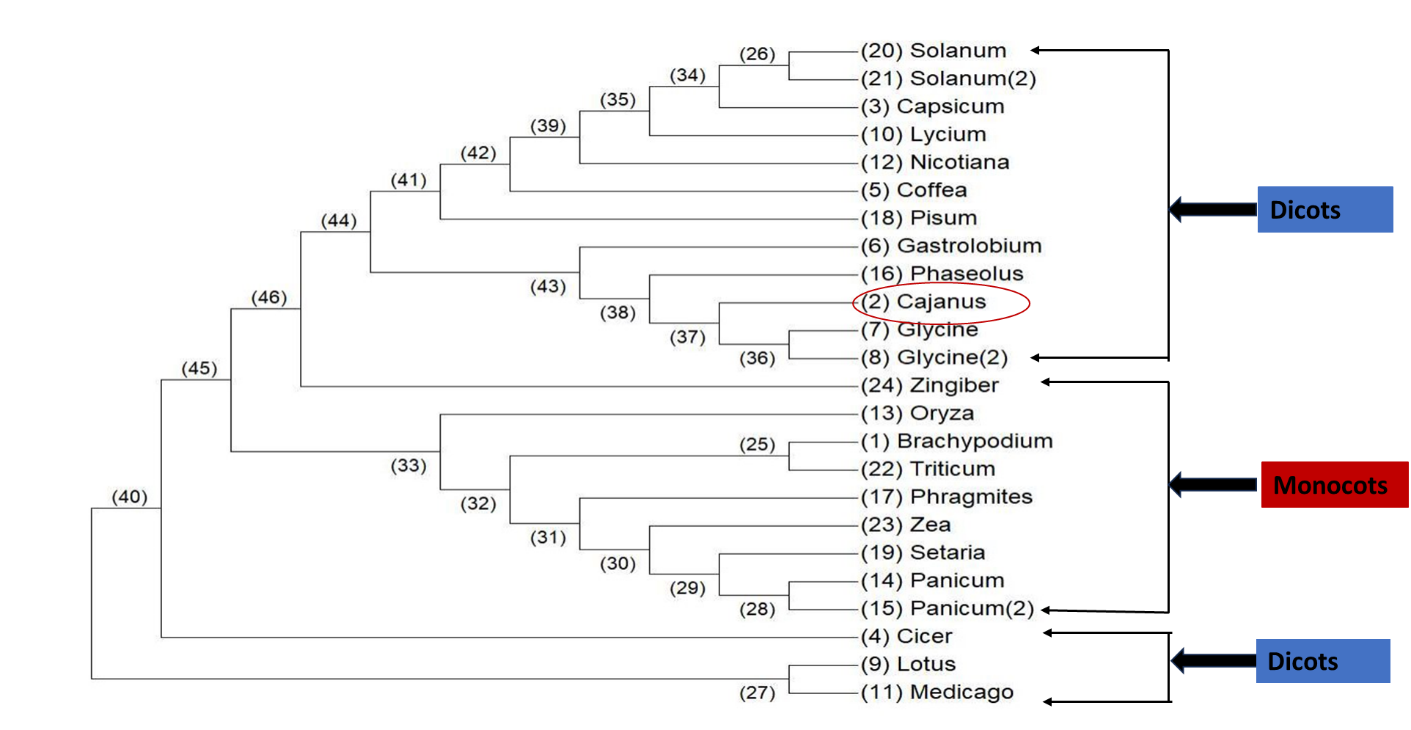


**Supplementary file 7: Figure S5.** Phylogenetic analysis and sequence alignment indicated conserved sequences, which were utilized for designing a single guide RNA.


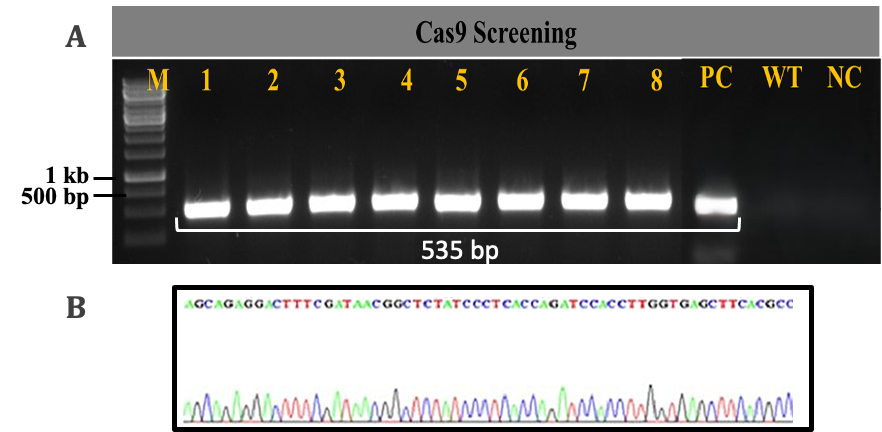


**Supplementary file 7: Figure S6.** Molecular characterization of putative positive transformed lines **(A)** PCR amplification of 535 bp region of Cas 9 gene from NIC-TK2-pCRISPR/Cas9 expression cassette. lanes 1–8 represent putative transformed pigeonpea samples M 1 kb DNA ladder (Thermofisher), WT genomic DNA of untransformed pigeonpea plant, PC positive control (plasmid DNA template), NC negative control. **(B)** Validation of Cas9 gene in transformed pigeonpea lines via sanger’s sequencing. M 1 kb DNA ladder (Thermofisher), WT genomic DNA of untransformed pigeonpea plant, PC positive control (plasmid DNA template), NC negative control. + represent edited lines; - non-edited (wild type).


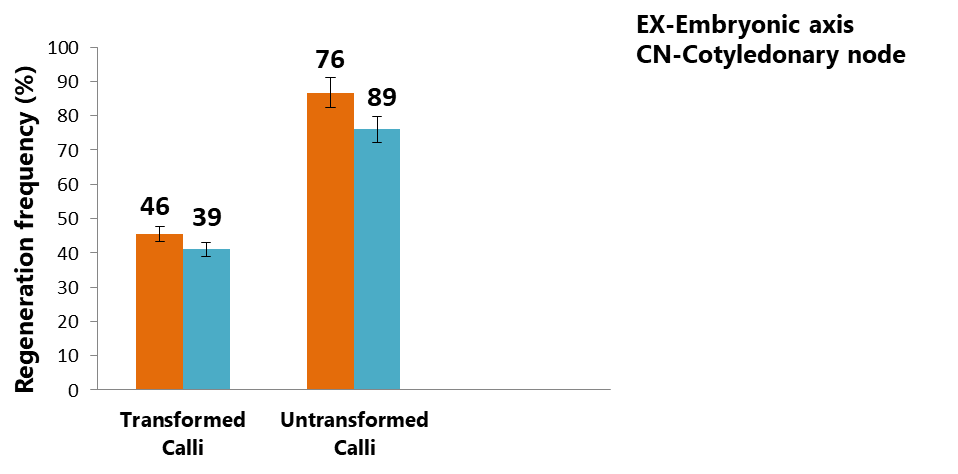


**Supplementary file 7: Figure S7.** Schematic representation of shoot regeneration frequency of transformed and untransformed calli from both the explants. Indigenously developed (NICTK2-pCRISPR/Cas9) were used to transformed the Cas9 gene.

**
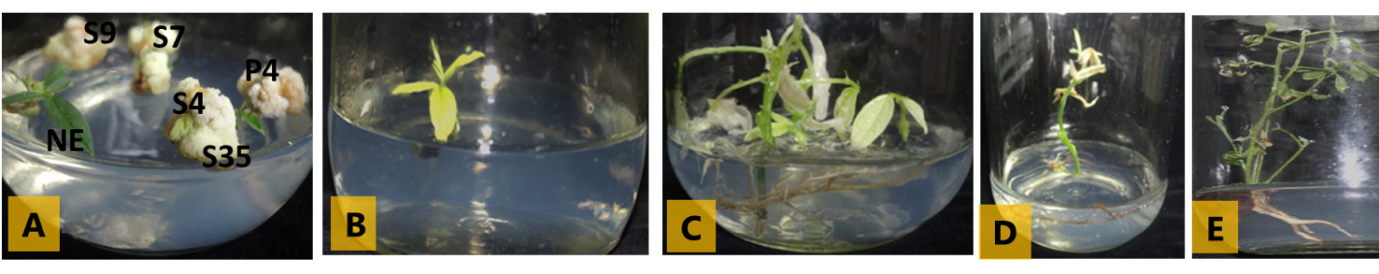
**

**Supplementary file 7: Figure S8.** Phenotypic variations observed in regenerated plants from different callus. **(A)** Regenerated shoots showing phenotypic variations, viz. pale green (regenerated shoot# P4), partial albino (regenerated from # S4, #S35), full albino (regenerated from #S7, #S9), non- edited (NE). **(B)** Regenerated shoots showing pale green phenotype**. (C)** Partial albino showing a mixture of green and white shoot tissues. **(D)** Fully albino **(E)** Non-edited plants.


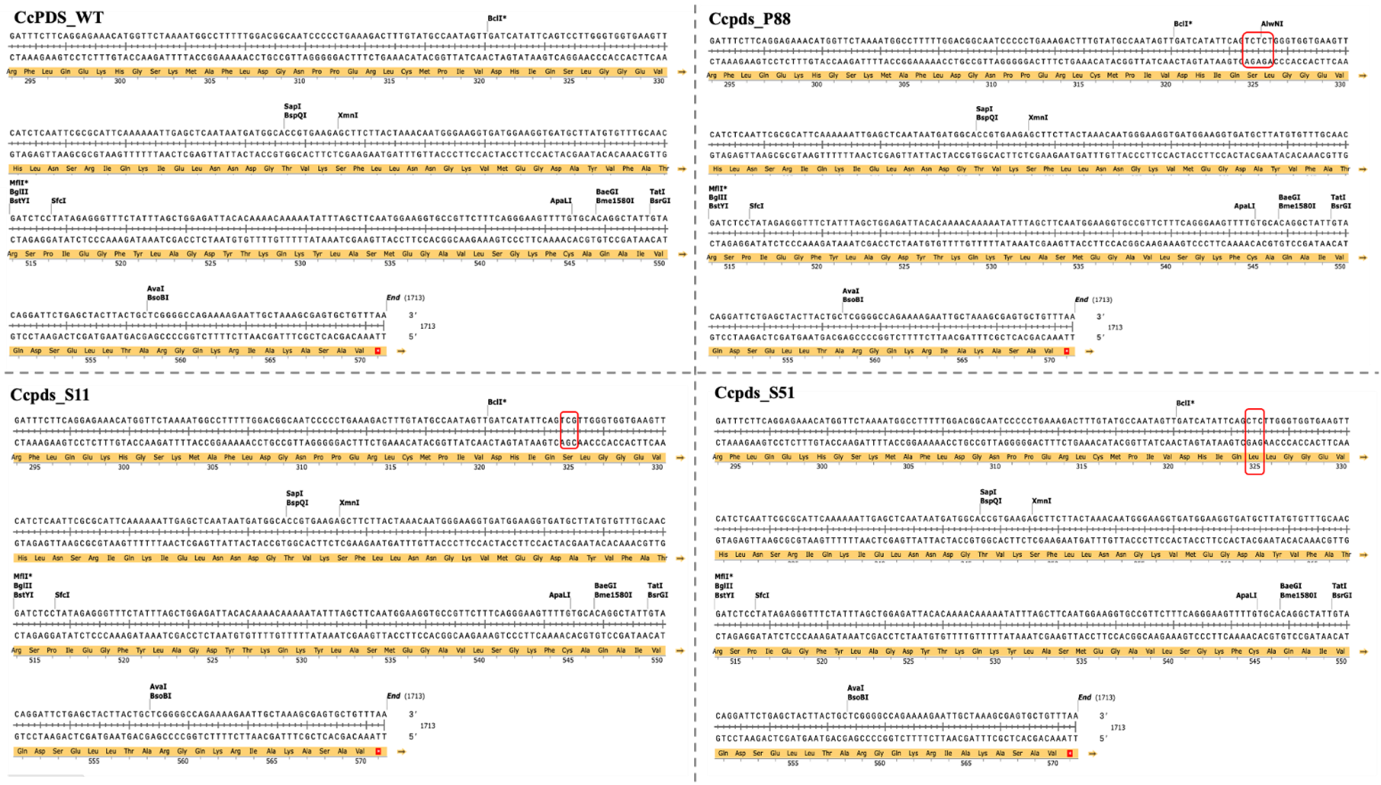


**Supplementary file 7: Figure S9.** Protein sequence of wild type CcPDS and three mutant plants showing green and dwarf morphology, where CcpdsP88 and CcpdsS11 shows changes at nucleotide level but same protein sequence, while CcpdsS51 shows nucleotide and protein sequence modification but still shows wild type morphology. The changes have been highlighted with red boxes.


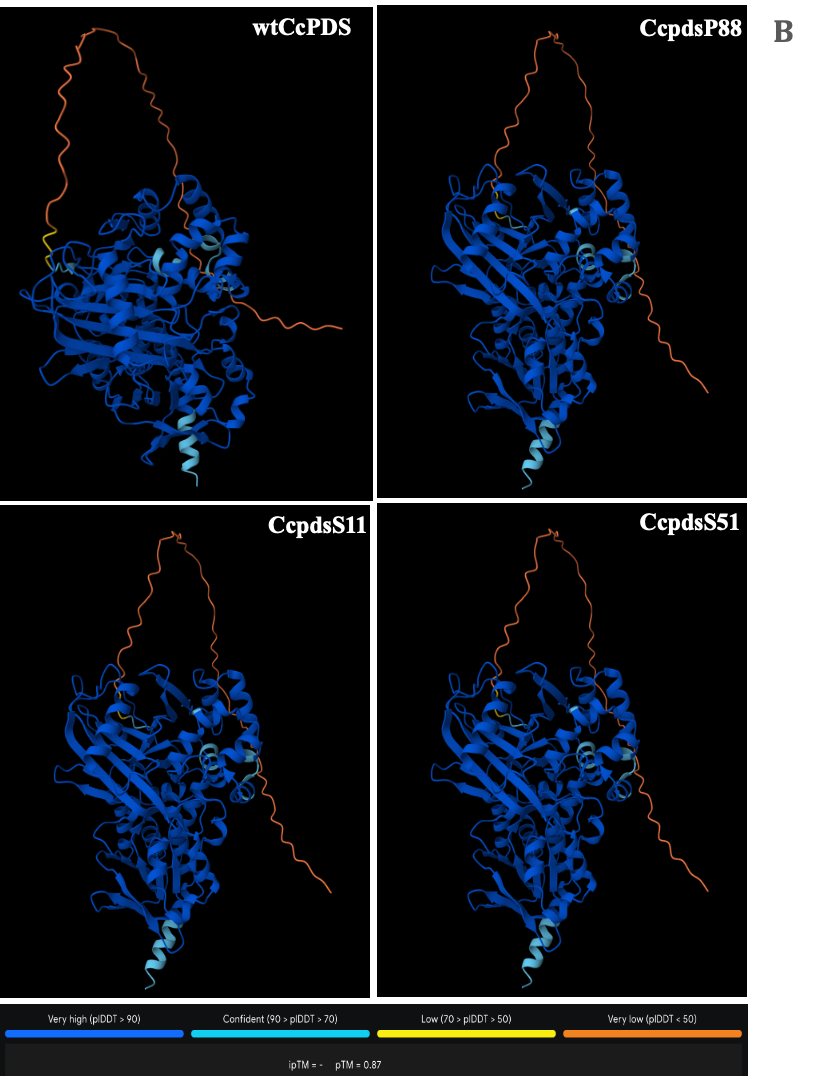


**Supplementary file 7: Figure S10.** Protein structure prediction analysis through Alphafold2 predicted (with 87% confidence) structure of WtCcPDS, CcpdsP88, CcpdsS11, and CcpdsS51protein.
